# Application of Temporally Controlled Release Systems in Periodontal Tissue Regeneration: From Material Design to Therapeutic Strategies

**DOI:** 10.3390/pharmaceutics18080927

**Published:** 2026-07-28

**Authors:** Ruohuai Zhang, Yuning Zeng, Lu Lin, Lei Jin, Dongfang Li

**Affiliations:** 1 School of Stomatology, Jiangxi Medical College, Nanchang University, Nanchang 330006, China; 413009250043@email.ncu.edu.cn (R.Z.); 413009240062@email.ncu.edu.cn (Y.Z.); linlu82@163.com (L.L.); 2 Jiangxi Provincial Key Laboratory of Oral Diseases, Nanchang 330006, China; 3 Jiangxi Provincial Clinical Research Center for Oral Diseases, Nanchang 330006, China; 4 Department of Stomatology, Taizhou Hospital, Wenzhou Medical University, Taizhou 317000, China; jl133252@163.com

**Keywords:** temporally controlled release, periodontal tissue regeneration, intelligent biomaterials, drug delivery, tissue engineering, periodontitis

## Abstract

Periodontitis, a chronic inflammatory disease driven by plaque biofilm, is a leading cause of tooth loss in adults worldwide. Effective treatment requires not only infection and inflammation control but, more critically, functional regeneration of the periodontal ligament, cementum, and alveolar bone. Periodontal regeneration, however, is a highly ordered, multi-stage biological cascade involving temporally coordinated phases of blood clot formation, inflammatory regulation, tissue formation, and remodeling. Conventional single-drug or mixed-delivery strategies cannot distinguish the distinct demands of each healing phase and fail to replicate this natural rhythm. Sequential controlled-release systems address this gap by delivering multiple bioactive agents (antimicrobials, immunomodulators, and growth factors) in a programmed order tailored to the healing cascade, enabling precise modulation of the periodontal microenvironment and orderly tissue regeneration. This review systematically summarizes advances in these systems, classifying material platforms into four categories: (1) diffusion-barrier and degradation-kinetics systems, including multilayer films, core–shell fibers, porous microspheres, and microneedle arrays; (2) stimuli-responsive systems triggered by pH, matrix metalloproteinases, reactive oxygen species, or exogenous physical stimuli; (3) cell and extracellular vesicle-based systems exploiting the inflammatory tropism of M2 macrophage-derived exosomes for targeted immune reprogramming; and (4) asymmetric structural designs achieving spatiotemporal coordination of physical and biochemical signals through hierarchical architectures. These systems follow an anti-infection/anti-inflammation first, osteogenesis later therapeutic logic, circumventing temporal antagonism among bioactive factors. However, significant challenges hinder clinical translation, including individualized prediction of release kinetics, long-term biocompatibility of carrier materials, material retention under dynamic oral conditions, translational limitations of animal models, and precise regulation of complex factor networks. Future progress will likely depend on multi-responsive and logic-gated systems, deeper integration of biotechnology and immunomodulation, personalized precision medicine, AI-driven material design, and robust clinical translational research.

## 1. Introduction

Periodontitis is a chronic disease initiated by plaque biofilm and involving host immune-inflammatory responses, characterized by periodontal attachment loss and progressive alveolar bone destruction, and it represents one of the leading causes of tooth loss in adults worldwide [1,2]. Successful periodontal treatment not only demands effective elimination of pathogenic bacteria and control of inflammation [3], but the more critical challenge lies in reversing the lost tissue architecture and achieving functional regeneration of the periodontal ligament, cementum, and alveolar bone [4].

The healing process of periodontal tissues, while analogous to general wound healing, is considerably more complex [5] and follows a finely orchestrated temporal cascade of events [4]: (I) Inflammatory phase—the initial stage requires rapid clearance of residual bacteria and appropriate modulation of the immune response to establish a microenvironment with a low bacterial load and resolved inflammation that is conducive to regeneration. (II) Proliferative phase—subsequently, periodontal ligament stem cells, fibroblasts, and osteoblasts must be recruited and their proliferation promoted to form new soft tissue attachment and bone matrix [6]. (III) Maturation and remodeling phase—the final stage involves guiding the orderly arrangement and functionalization of the newly generated tissue, thereby restoring the tenacity of the periodontal ligament and the mechanical support of the alveolar bone.

Conventional local delivery strategies, such as mouthwashes and gels, and early-generation local products, such as fibers and chips, although capable of direct application to the affected site, rely on a static, single-burst drug release profile that is ill-suited to this dynamic, multi-phasic healing rhythm [7,8]. These systems often fail to achieve prolonged retention in the complex oral environment, and the lack of control over their release kinetics can lead to a temporal mismatch in the release of antimicrobials and growth factors, thereby failing to provide the appropriate biological signals for tissue repair at the correct time [9].

Consequently, the field of periodontal regeneration is undergoing a conceptual transition from simple local delivery to intelligent sequential control. By leveraging bioadhesive materials, nanotechnology, and stimuli-responsive polymers [10], next-generation drug delivery systems are designed to achieve enhanced retention within the periodontal pocket, and to enable the sequential and on-demand release of multiple bioactive agents—either by responding to microenvironmental cues (e.g., pH, specific enzymes) or through pre-programmed structural designs (e.g., multilayer films, core–shell structures) [7]. More recently, the intelligent concept of temporal control has been further extended to the structural design of materials, where physical barrier and biological communication functions are integrated in a temporally coordinated manner to achieve temporal synergy—a biomimetic strategy that recapitulates the natural healing sequence from molecular release to macroscopic architecture.

Several recent reviews have surveyed complementary aspects of periodontal drug delivery and regenerative biomaterials. Su et al. comprehensively examined smart stimuli-responsive systems—encompassing pH-, enzyme-, ROS-, and photo-responsive platforms—for antimicrobial, immunomodulatory, and regenerative applications in periodontitis [11]. Zuo et al. reviewed bioactive scaffolds integrating controlled active-ingredient delivery with pathologically responsive microenvironment modulation [12]. Sha et al. focused specifically on pH-responsive nanocarrier design for periodontitis treatment [13]. Nakajima et al. surveyed the broader landscape of local drug delivery strategies, from injectable hydrogels to implantable devices [14]. Fischer et al. provided comprehensive guidance on biomaterial selection, fabrication methods, and biological considerations for periodontal tissue engineering [15]. Kumar et al. conducted an umbrella review of systematic reviews, confirming the efficacy of advanced biomaterials while noting persistent methodological heterogeneity across primary studies [16]. These contributions have substantially advanced the field; however, a dedicated synthesis that employs temporal control—the programmed ordering of multiple therapeutic events along the healing timeline—as the primary organizing principle has been lacking. Specifically, no prior review has (i) systematically classified periodontal sequential release systems across the full mechanistic spectrum from passive diffusion barriers to cell-based biological carriers to asymmetric structural designs within a single framework, (ii) provided a cross-strategy comparative assessment explicitly anchored to preclinical evidence strength and manufacturing feasibility, or (iii) integrated manufacturing scalability, batch-to-batch reproducibility, and regulatory classification as explicit evaluation dimensions alongside materials design. The present review addresses these gaps. We classify four mechanistically distinct temporal control strategies, critically compare their evidence maturity and translational readiness, identify shared and strategy-specific challenges that must be overcome for clinical adoption, and outline a forward-looking roadmap in which advances in logic-gated multi-responsive materials, gene and nucleic acid therapeutics, closed-loop personalized delivery, and AI-driven computational design converge toward the goal of functionally meaningful periodontal regeneration. We propose that the key to this convergence lies in achieving molecular-to-macroscopic biomimetic temporal synergy—in which the material not only delivers bioactive agents in the correct sequence but also provides temporally coordinated physical barrier and biological communication functions that collectively orchestrate the entire healing cascade.

## 2. Design Principles and Implementation Strategies of Sequential Controlled-Release Systems

**Figure 1:**
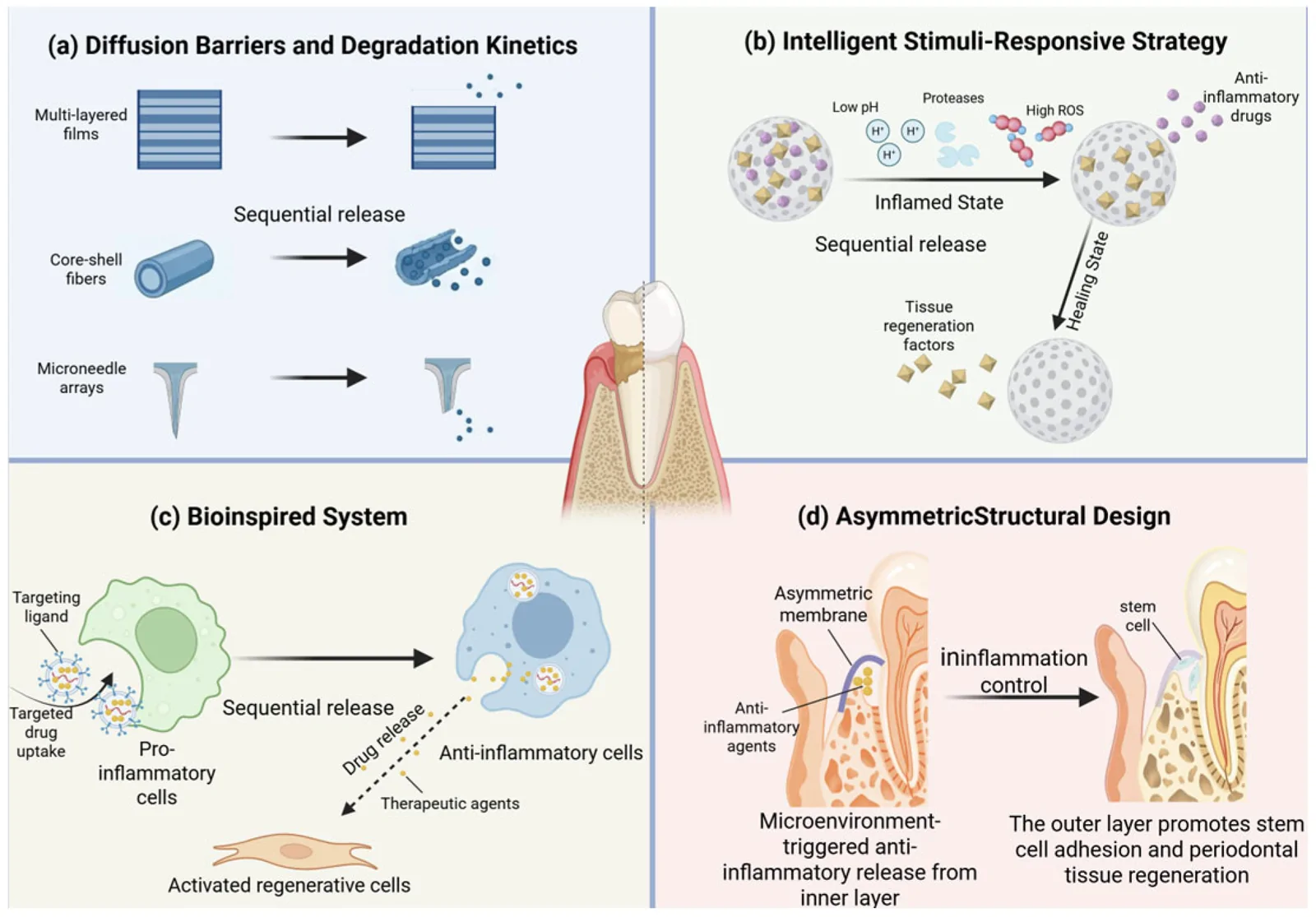
Major design strategies of sequential controlled-release systems for periodontal tissue regeneration: (**a**) Diffusion-barrier and degradation-kinetics strategy: sequential drug release is programmed through the predefined degradation or dissolution order of spatially segregated compartments such as multilayer films, core–shell fibers, porous microspheres, and microneedle arrays. (**b**) Intelligent stimuli-responsive strategy: release is triggered on-demand by sensing endogenous cues (e.g., low pH, elevated MMPs, excessive ROS) characteristic of the inflamed periodontal pocket, or by exogenous physical stimuli (e.g., NIR light). (**c**) Cell/extracellular vesicle-based strategy: engineered biological carriers exploit the natural inflammatory tropism of M2 macrophage-derived exosomes and stem cells to achieve targeted delivery and immune reprogramming. (**d**) Asymmetric structural design strategy: materials with inherent structural or functional asymmetry—such as bilayer membranes with spatially distinct physical/biological properties—exert temporally synergistic barrier, anti-inflammatory, and pro-regenerative functions across the healing cascade. Each of the four strategies is supported by multiple experimentally validated systems reviewed in Section 2.1, Section 2.2, Section 2.3 and Section 2.4, respectively. (The figure was created with BioRender.com).

The realization of sequential release hinges on exquisite material design. At present, the principal strategies can be classified into the following categories (Figure 1): sequential control based on diffusion barriers and degradation kinetics; intelligent sequential control based on stimuli-responsive materials; sequential control based on bioinspired systems; and temporal functional synergy based on asymmetric structural design.

### 2.1. Sequential Control Based on Diffusion Barriers and Degradation Kinetics

One of the most intuitive strategies for achieving sequential control is to engineer the physical structure [17] and degradation behavior [18] of materials to establish diffusion barriers and temporal release sequences for different drugs. Depending on the carrier morphology [19] and design dimensionality [20], this strategy is primarily realized through three structural configurations: film- and fiber-based systems with layered or core–shell architectures, functionalized microsphere systems built upon porous networks, and microneedle array systems. The design rationale and representative examples of each configuration are elaborated in the following subsections.

#### 2.1.1. Layered and Core–Shell Structured Systems

The core principle of this strategy lies in constructing release barriers through spatial segregation and differences in material properties [21,22]. By loading different drugs into distinct physically separated compartments and exploiting the differences among these compartments in degradation rate, permeability, or hydrophilicity/hydrophobicity, a predefined drug release order is achieved [23].

**Figure 2:**
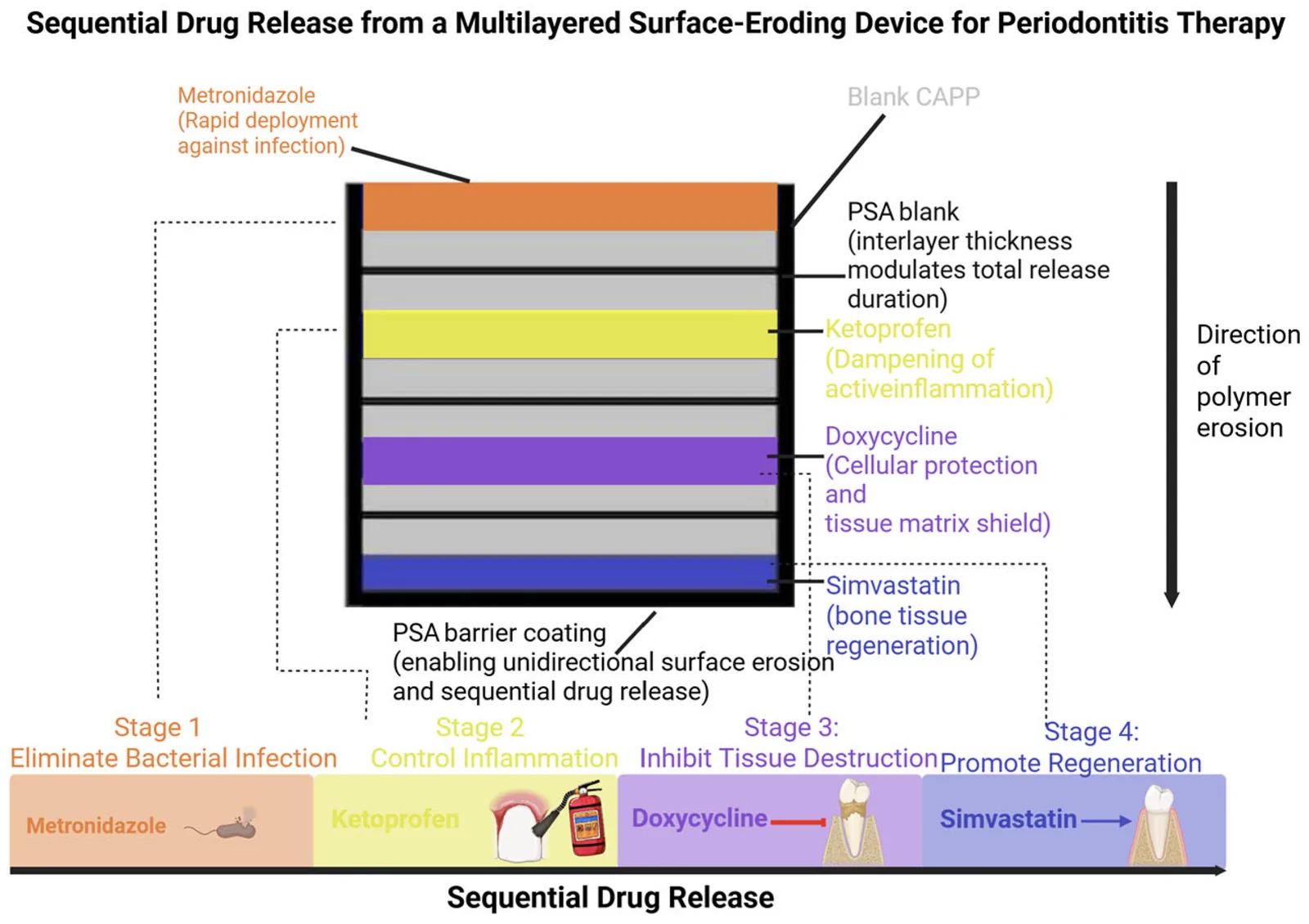
Structure and sequential release mechanism of the CAPP multilayer drug delivery system. The device comprises four drug-loaded cellulose acetate phthalate/Pluronic F-127 (CAPP) layers—metronidazole (antibacterial), ketoprofen (anti-inflammatory), doxycycline (anti-resorptive), and simvastatin (osteogenic)—interspersed with blank CAPP spacer layers. Thin poly(sebacic acid) (PSA) barrier layers were inserted between the blank layers to further prolong the erosion interval. The entire laminated stack is encapsulated by a PSA coating to ensure unidirectional surface erosion, such that the outermost layer is released first and the innermost layer last. This architecture produces a temporally programmed release sequence—antibacterial → anti-inflammatory → anti-resorptive → osteogenic—spanning approximately 160 h of total erosion. All four drugs retained near-complete bioactivity after encapsulation and release. The depicted release kinetics and bioactivity were demonstrated through in vitro erosion profiling and bioassays by Sundararaj et al. [25]; in vivo validation in a periodontal defect model has not been reported for this specific system. (The figure was created with BioRender.com).

At the macroscopic scale, layered stacking [24] represents a classic embodiment of this principle. The CAPP multilayer film delivery system (Figure 2), developed by Sundararaj et al., represents one of the earliest explorations of the sequential controlled-release concept in comprehensive periodontitis treatment [25]. The device was fabricated by solvent evaporation, laminating four drug-loaded cellulose acetate phthalate/Pluronic F-127 (CAPP) layers—metronidazole (antibacterial), ketoprofen (anti-inflammatory), doxycycline (anti-resorptive), and simvastatin (osteogenic)—interspersed with blank spacer layers, and the entire stack was encapsulated within a poly(sebacic acid) (PSA) coating to ensure unidirectional surface erosion. This design achieved a rigorously programmed temporal sequence governed entirely by material erosion: metronidazole in the outermost layer was released first within 15–25 h of incubation in PBS, eliminating infection during the initial erosion phase; ketoprofen release commenced subsequently and was completed between approximately 40 and 60 h to control inflammation; doxycycline release began at ~40 h, overlapping with the late ketoprofen phase, and continued through ~100 h to inhibit matrix metalloproteinase-mediated tissue destruction; and simvastatin, located in the innermost layer, was released during the final erosion phase to promote osteogenesis. Insertion of PSA barrier layers between the CAPP spacer layers extended the total device erosion time to 160 h, thereby prolonging the release intervals among the four drugs. Both metronidazole and ketoprofen retained essentially 100% of their bioactivity following encapsulation and release, confirming that the fabrication process did not compromise drug potency. This multilayered delivery system achieves a programmed sequential release: antibacterial and anti-inflammatory agents are released first, followed by anti-resorptive and osteogenic drugs, entirely through controlled erosion of the polymer layers. However, it should be noted that this study was limited to in vitro release characterization and bioactivity testing; no in vivo periodontal defect model was reported, leaving the translational relevance of this multilayer approach unvalidated in a physiologically relevant environment.

Beyond macroscopic layered stacking, researchers have also introduced the logic of sequential control into the fabrication of individual fibers, achieving programmed release through the design of core–shell microstructures. Liu et al. [26] developed a core–shell micelle-in-nanofiber membrane tailored to the dynamic progression of periodontitis, employing a one-step coaxial electrospinning strategy. SP600125, a c-Jun N-terminal kinase (JNK) inhibitor, was first encapsulated within poly(ethylene glycol)-block-poly(ε-caprolactone) (PEG-b-PCL) micelles (51.04 ± 12.24 nm in diameter), which were subsequently blended with gelatin to constitute the shell layer of the electrospun fibers; simultaneously, the osteogenic growth factor BMP-2 was loaded into the poly(vinyl alcohol) (PVA) fiber core. This spatial segregation enabled precisely time-programmed dual release: ~41–47% of SP600125 was released from the shell within the first 4 days (initial burst phase), with cumulative release exceeding 75% by day 28 for both the 10-SP-PMs and 20-SP-PMs formulations. In contrast, BMP-2 release from the core was not detected until day 12, after which its concentration gradually increased, reaching approximately 100 ng/mL by day 16 and being maintained at this level until day 22. In vitro, the early release of SP600125 effectively suppressed LPS-induced expression of pro-inflammatory cytokines (IL-1β, IL-6) and matrix metalloproteinases (MMP-2, MMP-9) in periodontal ligament cells (PDLCs), thereby clearing the inflammatory barrier for subsequent tissue repair. During the later phase, the sustained BMP-2 release upregulated osteogenic markers at both gene and protein levels (ALP and Runx2 at day 14; OPN and OCN at day 21) [20]. In vivo, in a beagle dog class II furcation defect model (5 mm diameter × 2 mm depth), this membrane served as a single-step therapeutic intervention—applied only once to the lesion site—and within two months markedly inhibited alveolar bone destruction while achieving substantial bone defect repair, as assessed by micro-CT (BV/TV quantification) and sequential fluorochrome labeling. These results provide early proof-of-concept that sequential controlled-release strategies can achieve both anti-inflammation and bone regeneration through a single, surgically convenient intervention in a clinically relevant large-animal model.

Jiang et al. [27] developed a core–shell electrospun scaffold based on poly(glycolic acid)–silk fibroin (PGA-SF), in which the anti-inflammatory cytokine IL-4 was physically adsorbed onto the SF shell and osteoconductive tricalcium phosphate (TCP) nanoparticles were encapsulated within the PGA core. This spatial segregation enabled a dual-mode sequential release profile: >70% of IL-4 was released within the first 3 days, establishing a rapid immunomodulatory phase, followed by slow, continuous release over at least 7 days; concurrently, Ca^2+^ release from TCP in the PGA core peaked at day 7–10 and remained detectable for up to 46 days, steadily supplying calcium and phosphate ions throughout the middle and late phases of healing. The optimal IL-4 loading was determined to be 156.55 ng/cm^2^ (at a soaking concentration of 300 ng/mL). In vitro, in a transwell co-culture system of RAW 264.7 macrophages and BMSCs, the PGA/TCP-SF/IL-4 scaffold significantly upregulated M2 polarization markers (Arg-1, CD206) and downregulated M1-associated pro-inflammatory mediators (TNF-α, iNOS, IL-1β), thereby establishing a favorable anti-inflammatory microenvironment at the initial stage. During the subsequent phase, the sustained TCP release enhanced ALP activity, mineralization (Alizarin Red S), and osteogenic protein expression (RUNX2, OCN) in BMSCs. In vivo, implantation in a rat critical-size calvarial defect model demonstrated markedly superior bone regeneration compared with single-factor groups, with micro-CT quantification revealing elevated BV/TV and trabecular number (Tb.N) and decreased trabecular spacing (Tb.Sp). Immunofluorescence analysis at 2 weeks post-implantation confirmed enhanced infiltration of CD206^+^ M2 reparative macrophages and reduced presence of iNOS^+^ M1 pro-inflammatory macrophages in the defect region. This dual-mode sequential release strategy—immunomodulation first, followed by sustained osteogenesis—synergistically orchestrated the osteoimmune microenvironment, demonstrating that temporally coordinated modulation of macrophage polarization and osteogenic differentiation can yield regenerative outcomes superior to those achievable with either cue alone.

In a notable extension, surface engineering modifications can be superimposed onto core–shell structures to construct multiple release pathways within a single carrier. Cheng et al. [28] designed a composite nanofibrous membrane that uniquely integrates coaxial electrospinning with layer-by-layer (LbL) self-assembly to expand the complexity of sequential release programs. Specifically, a silk fibroin/polycaprolactone (SF/PCL) composite served as the shell and poly(vinyl alcohol) (PVA) as the core of the electrospun fibers, in which the osteogenic factor BMP-2 was encapsulated. Connective tissue growth factor (CTGF) was subsequently deposited onto the fiber surface via LbL electrostatic adsorption of chitosan–CTGF bilayers. Under the optimized formulation (SF/PCL ratio of 1:5; 20 LbL bilayers), this architecture achieved a clear temporal separation of the two factors: CTGF exhibited an initial burst release on day 1 that was maintained for approximately 5 days, with release largely confined to the first 10 days and a cumulative release of 90 ± 4% over 40 days, while BMP-2 displayed a sustained, linear release profile over the entire 30-day incubation period with minimal initial burst. Notably, LbL deposition served a dual function—it not only provided the CTGF reservoir on the fiber surface but also suppressed the initial BMP-2 burst from the core, as partial BMP-2 loss during the coating process converted the core release into a more linear, sustained mode. In vivo tracking confirmed these distinct temporal profiles: fluorescently labeled BMP-2 signal persisted at the implant site for 30 days, whereas CTGF signal became undetectable by 15 days post-implantation. In a mouse calvarial defect model, this sequential dual-delivery system—an early angiogenic/chemotactic CTGF signal followed by a sustained osteogenic BMP-2 signal, faithfully recapitulating the natural temporal expression pattern of these growth factors during bone healing—improved bone regeneration by 43% compared with single BMP-2 delivery, as quantified by micro-CT analysis. By demonstrating that independent spatial segregation strategies (coaxial electrospinning and LbL assembly) can be superimposed within a single construct, this work illustrates a generalizable design principle for constructing multi-factor sequential release systems with expanded temporal complexity.

#### 2.1.2. Functionalized Porous Microsphere Systems

Functionalized porous microspheres are micrometer-sized spherical carriers constructed from biodegradable polymers such as poly(lactic-co-glycolic acid) (PLGA) and gelatin [29], and they feature an internal three-dimensional, interconnected porous network [30]. By virtue of their high specific surface area [31], excellent cell-carrying capacity, and suitability for injectable delivery [32], functionalized porous microsphere systems offer distinct advantages in local periodontal therapy. The central design logic for achieving sequential control in these systems lies in the synergistic programming of spatial segregation and release kinetics: different bioactive factors are differentially loaded onto the surface coating, within the pore interfaces, or inside the matrix of the microspheres. The differences in diffusional resistance dictated by their spatial locations [33] are then exploited to precisely prescribe the release sequence and rate of multiple factors [34].

Luo et al. [35] developed calcitonin gene-related peptide (CGRP)-loaded porous PLGA/polydopamine microspheres (PMs) for the co-delivery of bone marrow mesenchymal stem cells (BMSCs), realizing a temporally orchestrated therapeutic strategy in which the biological sequence—microenvironment modulation first, followed by stem cell-mediated regeneration—is programmed through the interplay of sustained drug release and cellular engraftment. The CGRP-loaded PMs achieved sustained release with a cumulative output of 6247.4 ± 44.7 pg by day 8 (loading capacity: ~0.35 ng CGRP per mg of microspheres). Upon implantation into the periodontitis lesion, this sustained CGRP release effectively promoted inflammation resolution and immune microenvironment remodeling, partly through inhibition of the ROS/NLRP3/caspase-1 signaling pathway and the consequent reduction in pro-inflammatory cytokines such as IL-1β [35,36]. This early anti-inflammatory intervention directly protected the co-delivered BMSCs from inflammation-induced apoptosis and functional impairment, markedly increasing the survival rate of the transplanted stem cells at the diseased site. Subsequently, the protected BMSCs were able to engraft, proliferate, and efficiently differentiate into osteoblasts within the improved microenvironment, while the porous architecture of the microspheres simultaneously provided physical protection and a three-dimensional niche for the transplanted cells. In vivo, in a mouse ligature-induced periodontitis model, micro-CT analysis demonstrated that the full combination (PM/CGRP/BMSCs) achieved the highest bone volume fraction (BV/TV = 84.07%), significantly exceeding PM/BMSCs alone (76.39%), PM/CGRP alone (55.83%), and PBS control (37.18%); bone mineral density (BMD) followed the same trend, reaching 1003.16 ± 76.53 mgHA/cm^3^ in the PM/CGRP/BMSCs group versus 807.57 ± 56.90 mgHA/cm^3^ in the PBS control. These results highlight the considerable potential of sequential controlled-release to sustain the success of stem cell-based therapies under challenging inflammatory conditions: CGRP-mediated microenvironment conditioning is a prerequisite for—rather than merely an adjunct to—effective BMSC-mediated regeneration.

**Figure 3:**
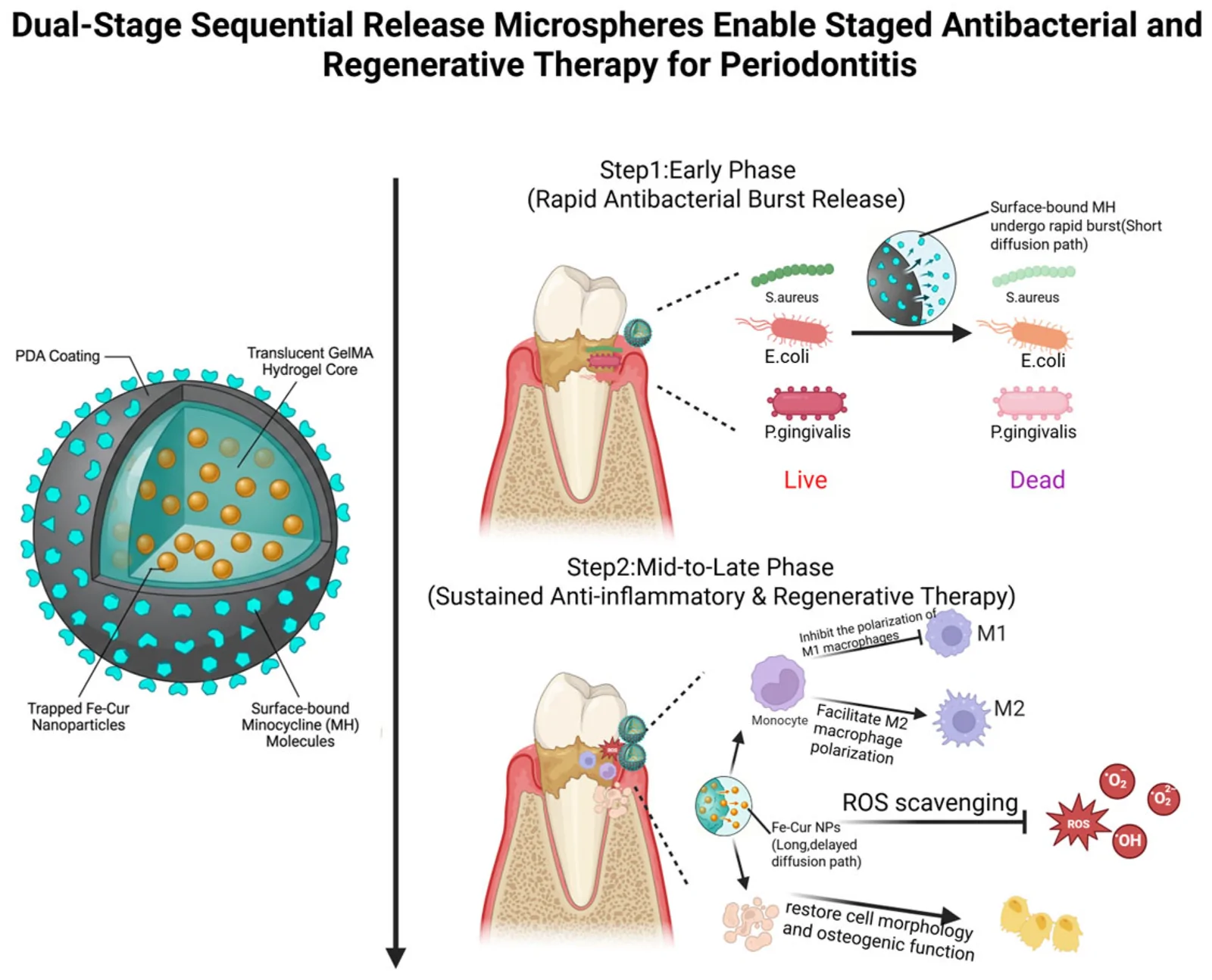
Design and sequential therapeutic mechanism of the GM@Fe-Cur/PDA/MH multifunctional injectable microsphere system. GelMA-based microspheres, fabricated by microfluidic technology, incorporate two spatially segregated drug-loading domains: minocycline hydrochloride (MH) is adsorbed onto the polydopamine (PDA) coating surface via electrostatic and π–π interactions for rapid early release (≈58% within 96 h), while iron-curcumin nanoparticles (Fe-Cur NPs) are encapsulated within the GelMA matrix for sustained, delayed release (≈7% at 96 h). The PDA coating additionally provides a photothermal antibacterial function under NIR irradiation. This spatial segregation automatically programs the therapeutic sequence—antibacterial action (rapid MH release + photothermal effect) → antioxidant/anti-inflammatory modulation → osteogenesis (sustained Fe-Cur NP release). The depicted release kinetics, antibacterial activity, antioxidant effects, and macrophage polarization modulation were experimentally demonstrated in vitro; in vivo efficacy in reducing alveolar bone loss was confirmed by micro-CT analysis in a rat ligature-induced periodontitis model [37]. (The figure was created with BioRender.com).

Beyond the single-factor modulation of the microenvironment, considerable effort has been devoted to integrating multiple functions—including antibacterial, antioxidant, anti-inflammatory, and osteogenesis-promoting activities—into a single microsphere platform, where the temporal expression of these functions is programmed through exquisite physical structural design. A representative example of this direction is the multifunctional injectable microsphere system (GM@Fe-Cur/PDA/MH) developed by Xiu et al. [37] (Figure 3).

In this system, GelMA-based microspheres were fabricated by microfluidic technology, and a differentiated loading strategy was employed: the antibacterial agent minocycline hydrochloride (MH) was adsorbed onto the polydopamine (PDA) coating surface via electrostatic and π–π interactions, while iron-curcumin nanoparticles (Fe-Cur NPs), possessing both antioxidant and osteogenesis-promoting potential, were encapsulated within the GelMA matrix. This spatial compartmentalization dictated the release kinetics: MH, facing minimal diffusion resistance from its surface-loaded configuration, achieved 57.80% cumulative release within 96 h under physiological conditions (60.08% in an LPS-containing environment that partially accelerated surface degradation), swiftly establishing an effective local antibacterial concentration at the early treatment stage. In contrast, Fe-Cur NPs, physically hindered by the microsphere matrix and its slow degradation, exhibited markedly delayed release kinetics, with only 6.96% cumulative release at 96 h (7.28% in LPS). This approximately 8-fold difference in early release rates ensured that the antibacterial phase was temporally decoupled from the subsequent antioxidant and osteogenic phases. The PDA coating further contributed a photothermal antibacterial function under NIR irradiation, synergistically enhancing the early bactericidal effect. During the middle and late phases of healing, the sustained release of Fe-Cur NPs continuously scavenged reactive oxygen species, modulated macrophage polarization, and promoted osteogenic signaling, with mechanistic studies implicating activation of the Nrf2 signaling pathway and inhibition of NLRP3 inflammasome activation. In vivo, in a rat ligature-induced periodontitis model, micro-CT analysis of the cementoenamel junction-to-alveolar bone crest (CEJ-ABC) distance demonstrated that GM@Fe-Cur/PDA/MH treatment significantly reduced alveolar bone loss compared with untreated periodontitis controls. This system illustrates a generalizable design principle: by engineering differential diffusion barriers through surface loading versus matrix encapsulation within a single carrier, a pre-programmed therapeutic sequence—antibacterial action first, followed by antioxidant/anti-inflammatory modulation and osteogenesis—can be achieved without relying on external triggers or multi-component assembly.

#### 2.1.3. Microneedle Array Systems

Microneedles (MNs) are array devices composed of micrometer-sized needle structures, tens to hundreds of micrometers in length, arranged on a supporting baseplate [38]. By virtue of their minimally invasive [39], pain-free [40], and barrier-penetrating capabilities [41], MN systems offer distinct advantages for local drug delivery in periodontal therapy [42]. Microneedle arrays can penetrate the gingival epithelium or the inner wall of the periodontal pocket, delivering drugs loaded in the needle tips directly into the deep lesion site [43] and thereby enabling precise and programmed release [44]. The core design principle underpinning their use as a sequential control platform lies in constructing a delivery system with spatial segregation and temporally controlled release by exploiting the physical separation between the needle body and the baseplate [45], differences in material degradation kinetics across distinct regions [46], and hierarchical drug-loading architectures [47]. The specific mechanisms can be summarized into three categories. (1) Bilayer/multilayer structures, in which different drugs are separately loaded into a rapidly dissolving baseplate and a slowly degrading needle body, realizing a sequential program of rapid basal release followed by sustained release from the needles [48]. (2) Core–shell structures, analogous to core–shell fibers, in which drugs are encapsulated within distinct regions of the needle, with the degradation rate of the shell material dictating the release timing of the core payload [49]. (3) Hierarchical carrier-in-microneedle systems, in which drug-loaded nanoparticles or microparticles are further embedded within the needle body, enabling finer multi-stage release through differential degradation kinetics of the constituent carriers [47].

Zhang et al. [50] designed a modular immunomodulatory microneedle (MN) patch consisting of a rapidly dissolving gelatin baseplate and biodegradable GelMA microneedles, thereby constructing a bilayer system with three-tier release logic. The baseplate was loaded with tetracycline hydrochloride and dissolved within approximately 10 min of implantation, delivering an immediate burst of antibiotic to rapidly reduce the local bacterial burden. The crosslinked GelMA needle layer was embedded with two distinct carrier types for sustained release: poly(lactic-co-glycolic acid) (PLGA) nanoparticles (~150 nm) loaded with tetracycline for prolonged antibacterial protection, and heparin-functionalized mesoporous silica microparticles (SiMPs, ~15 μm diameter, ~10 nm pore size) loaded with IL-4 and TGF-β for immunomodulation. The heparin modification served a dual purpose—enhancing cytokine loading via electrostatic interactions with the positively charged IL-4 (pI 9.17) and TGF-β (pI 8.83), and protecting these proteins from denaturation during MN fabrication. The SiMPs degraded over approximately two weeks in vitro, providing sustained cytokine release throughout this period. In vitro, the antibiotic release completely inhibited bacterial growth; the sustained release of IL-4 and TGF-β significantly suppressed pro-inflammatory gene expression (iNOS, IL-1β) while upregulating M2 macrophage markers (MRC1, ARG-1), and induced regulatory T cell (Treg) formation more effectively than soluble cytokine administration. In vivo, in a rat periodontitis model, this modular delivery strategy suppressed pro-inflammatory factors and promoted pro-regenerative signals and tissue healing. By partitioning three distinct release modalities—immediate burst (gelatin baseplate), sustained nanoparticle-based release (PLGA NPs), and prolonged heparin-mediated cytokine delivery (SiMPs)—within a single patch, this work demonstrates a modular approach to constructing multi-rate delivery systems without requiring complex multi-step carrier fabrication.

Within the MN array platform, the core–shell architecture offers an elegant design for achieving sequential control. A representative example of this direction is the matrix metalloproteinase (MMP)/pH dual-responsive core–shell microneedle system constructed by Liu et al. [44]. The shell consists of a gelatin methacryloyl (GelMA)/silk fibroin methacryloyl (SilMA) double-network hydrogel incorporating MMP-sensitive peptide sequences and loaded with glycyrrhizic acid-functionalized MIL-101 metal–organic frameworks (GA-MOF), which possess anti-inflammatory and antioxidant functions. The core comprises mesenchymal stem cell-derived exosomes (MSC-Exo) encapsulated within a SilMA hydrogel. In the periodontitis microenvironment—characterized by elevated MMP-9 and an acidic pH of approximately 6.5—the MMP-sensitive peptide crosslinks in the shell are preferentially cleaved, triggering rapid GA-MOF release over the initial several days to scavenge reactive oxygen species, suppress the pro-inflammatory cytokine storm (downregulating TNF-α, IL-6, IL-1β, and MMP-9), and create a favorable niche for subsequent regenerative signaling. Thereafter, exosomes are continuously released from the core over several weeks, inducing macrophage polarization toward the M2 phenotype (CD206^+^) and promoting osteogenic differentiation of BMSCs, as evidenced by a 2.3-fold increase in ALP-positive osteogenic activity relative to untreated controls. Micro-CT and histological analyses at day 28 in a rat periodontitis model demonstrated that EGGS MNs significantly reduced the CEJ–ABC distance, increased bone volume fraction (BV/TV) and trabecular thickness (Tb.Th), and upregulated osteogenic markers including RUNX2 and OPN. Through the integration of core–shell spatial partitioning with a dual-responsive release mechanism, this study exemplifies how an “anti-inflammation-first, osteogenesis-later” strategy can be realized via MN-based sequential therapy for periodontal regeneration.

**Figure 4:**
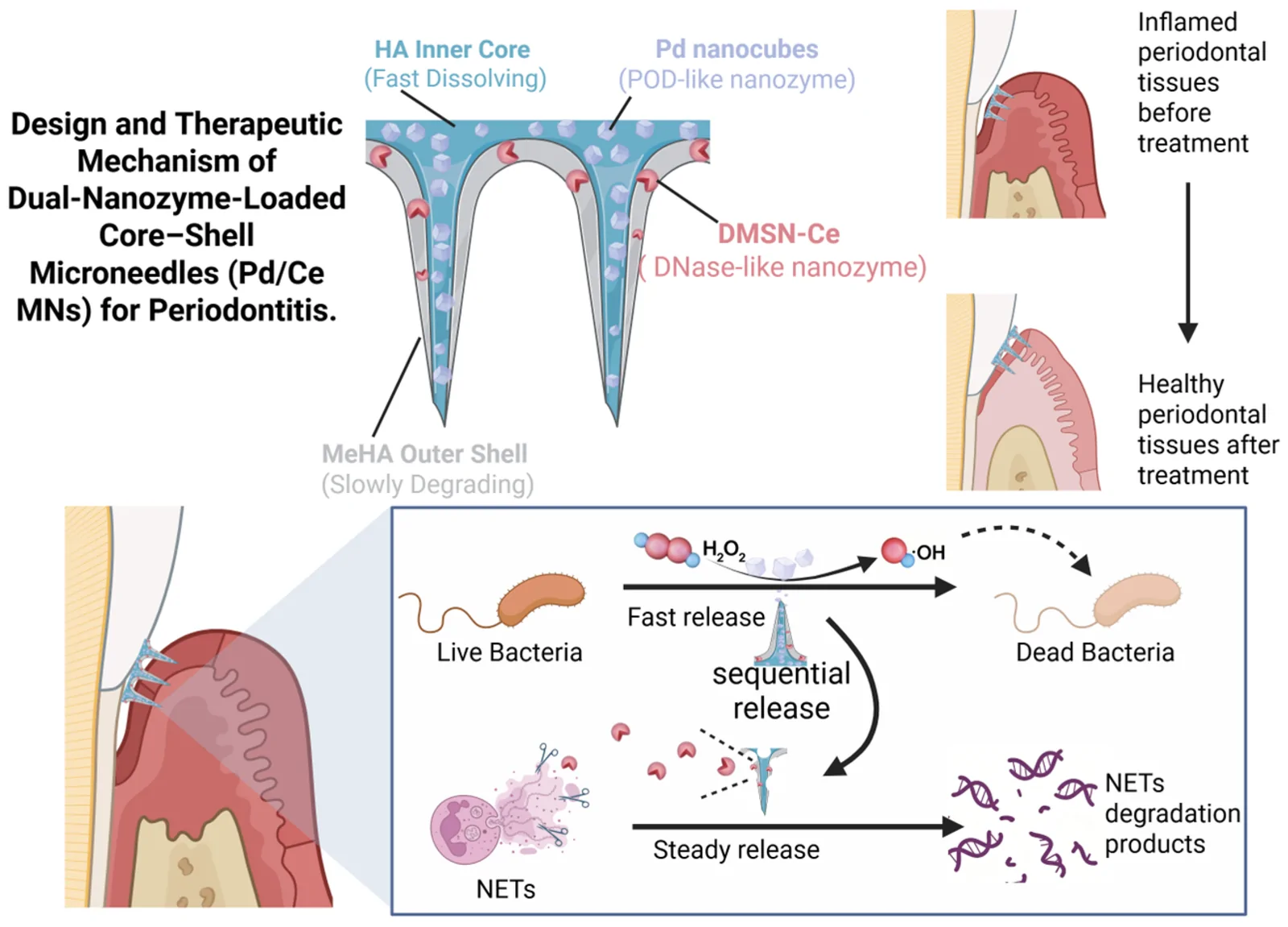
Design and sequential therapeutic mechanism of the Pd/Ce MNs dual-nanozyme core–shell microneedle system. The hyaluronic acid (HA) inner core encapsulates Pd nanocubes with peroxidase (POD)-like activity, while the methacrylated hyaluronic acid (MeHA) outer shell is loaded with dendritic mesoporous silica–cerium nanozymes (DMSN-Ce) possessing DNase-like activity. Upon implantation, differential degradation kinetics program the therapeutic sequence: rapid dissolution of the HA core enables immediate Pd-catalyzed conversion of endogenous H_2_O_2_ to bactericidal hydroxyl radicals (·OH), while the slower degradation of the crosslinked MeHA shell provides sustained release of DMSN-Ce nanozymes that hydrolyze the DNA backbone of neutrophil extracellular traps (NETs), thereby suppressing NETs-driven inflammatory tissue destruction. Release kinetics were characterized in vitro (Pd: 89.5% within 5 h; DMSN-Ce: 87.4% over 7 days); in vivo antibacterial efficacy, NETs degradation, and attenuation of alveolar bone resorption were confirmed in a rat ligature-induced periodontitis model [51]. (The figure was created with BioRender.com).

Artificial nanozymes have also been incorporated into core–shell microneedles to synergistically achieve antibacterial action and immunomodulation. Shan et al. [51] designed a dual-nanozyme-loaded core–shell microneedle system (Pd/Ce MNs) (Figure 4). The core consisted of hyaluronic acid (HA) encapsulating Pd nanocubes with peroxidase (POD)-like activity, while the shell was composed of methacrylated hyaluronic acid (MeHA) loaded with dendritic mesoporous silica-cerium nanozymes (DMSN-Ce) possessing DNase-like activity. The design logic exploited differential degradation kinetics: rapid dissolution of the HA core upon implantation enabled immediate release of Pd nanozymes, which catalyze the conversion of endogenous H_2_O_2_ to bactericidal hydroxyl radicals (·OH) for potent local antibacterial action; in parallel, the slower degradation of the MeHA shell provided sustained release of DMSN-Ce nanozymes, which degrade neutrophil extracellular traps (NETs) through DNA hydrolysis, thereby suppressing the NETs-driven component of periodontal inflammatory tissue destruction. In a rat ligature-induced periodontitis model, Pd/Ce MNs significantly reduced both the local bacterial burden and NETs levels, with micro-CT and histological analyses confirming alleviated alveolar bone resorption. This study highlighted the advantages of dual-nanozyme synergistic therapy and expanded the application of core–shell microneedles in sequential antibacterial and immunomodulatory treatment for periodontitis.

#### 2.1.4. Comparative Assessment

A cross-platform comparison of the three diffusion barrier-based strategies reveals notable differences in both evidence maturity and the fidelity with which sequential release logic is realized.

(1) Evidence strength: Among the nine representative systems surveyed in this section, only one—the core–shell micelle-in-nanofiber membrane developed by Liu et al. [26]—has been evaluated in a large-animal model (Beagle dog, Class II furcation defect), placing core–shell nanofiber membranes at the highest current evidence tier. The remaining systems—including all porous microsphere and microneedle platforms—have been tested exclusively in small-animal models (rat or mouse), and the CAPP multilayer film [25] has not progressed beyond in vitro characterization. Importantly, direct quantitative comparison of bone morphometric outcomes (e.g., BV/TV, CEJ-ABC distance) across these studies is methodologically unsound, given differences in animal species, defect type, observation time points, and region-of-interest definitions. Comparisons are therefore best anchored to evidence tier (large animal > small animal disease model > small animal surgical defect > in vitro only) rather than to numerical values.

(2) Sequential release fidelity: Most systems in this section successfully align their fast-release phase with antibacterial or anti-inflammatory therapy and their slow-release phase with osteogenesis, consistent with the biological sequence of periodontal healing. However, the degree of temporal separation between the two phases varies considerably across platforms. Systems employing discrete spatial compartments achieve the clearest phase separation: the Zhang et al. [50] immunomodulatory MN patch exemplifies this principle with three kinetically distinct tiers—gelatin baseplate dissolution within ~10 min, tetracycline release from PLGA nanoparticles over days, and cytokine release from heparin-functionalized SiMPs over approximately two weeks. Similarly, the Shan et al. [51] Pd/Ce MN system achieves a ~33-fold difference in release timescale between the rapidly dissolving HA core (Pd: 89.5% within 5 h) and the slowly degrading MeHA shell (DMSN-Ce: 87.4% over 7 days), and the Xiu et al. [37] microsphere system produces an ~8-fold difference in cumulative release at 96 h between surface-adsorbed MH and core-encapsulated Fe-Cur. In contrast, systems that rely solely on differential degradation within a single matrix—such as the CAPP multilayer film [25], which produces a continuous release spectrum spanning 15–120 h—exhibit substantial overlap between what are designated as sequential phases. The Liu et al. [26] core–shell nanofiber membrane achieves temporal separation through a biologically meaningful delay in BMP-2 release onset (~day 12) rather than through a large release-rate ratio, demonstrating that phase timing can be as important as phase separation.

(3) Translational readiness: Among the three diffusion-barrier strategies, microneedle array systems benefit from the most mature translational infrastructure: transdermal microneedle patches have established clinical manufacturing workflows, standardized quality control parameters (needle height uniformity, mechanical strength, dissolution kinetics), and an existing regulatory framework that can be adapted to periodontal indications [50,51]. Porous microsphere systems occupy an intermediate position—their injectable format is inherently compatible with existing syringe-based clinical delivery into the periodontal pocket, and microfluidic fabrication technologies are increasingly scalable, but batch-to-batch consistency in drug loading distribution across the sphere population remains a manufacturing challenge [35,37]. Core–shell nanofiber membranes, despite carrying the highest evidence tier (large-animal validation [26]), face the greatest translational hurdles: coaxial electrospinning is a multi-parameter process (voltage, flow rate, humidity, collector distance) whose reproducibility at manufacturing scale has not been demonstrated, and the surgical placement of a membrane requires flap reflection—a more invasive procedure than injection or patch application. Importantly, these relative rankings reflect current technological maturity rather than inherent potential; advances in manufacturing automation and minimally invasive deployment could alter the translational calculus.

A detailed discussion of manufacturing complexity, regulatory pathways, and clinical workflow integration common to all platforms is provided in Section 3.2.6 and Section 4.

### 2.2. Intelligent Sequential Control Based on Stimuli-Responsive Materials

Stimuli-responsive delivery systems are capable of sensing specific alterations in the pathological microenvironment within the periodontal pocket—such as changes in pH, enzyme levels, and reactive oxygen species (ROS) concentrations—and consequently triggering the sequential release of therapeutic agents [52], thereby enabling environment-responsive drug release [10].

#### 2.2.1. pH-Responsive Systems

During the healing process of periodontitis, the local pH is not constant but undergoes dynamic variations. In sites with active inflammation, the pH can drop to approximately 5.5 [53], whereas as inflammation resolves and tissue repair progresses, the pH gradually returns toward neutral [54]. This dynamic evolution of pH provides an ideal endogenous trigger for constructing sequential controlled-release systems [10]. Exploiting this characteristic, researchers have designed intelligent carriers that capitalize on pH fluctuations to achieve temporally programmed release of therapeutic agents. In the early stage of periodontitis (low-pH environment), the delivery system responds to the acidic milieu by preferentially releasing antimicrobial or anti-inflammatory drugs to rapidly control infection. Once the periodontitis is brought under control and the tissue enters the remodeling phase, the system helps to shape a microenvironment conducive to tissue regeneration [55]. This sequential logic—debridement first and repair thereafter—elegantly transforms the pathological change in the microenvironment into an executive switch for the therapeutic program.

**Figure 5:**
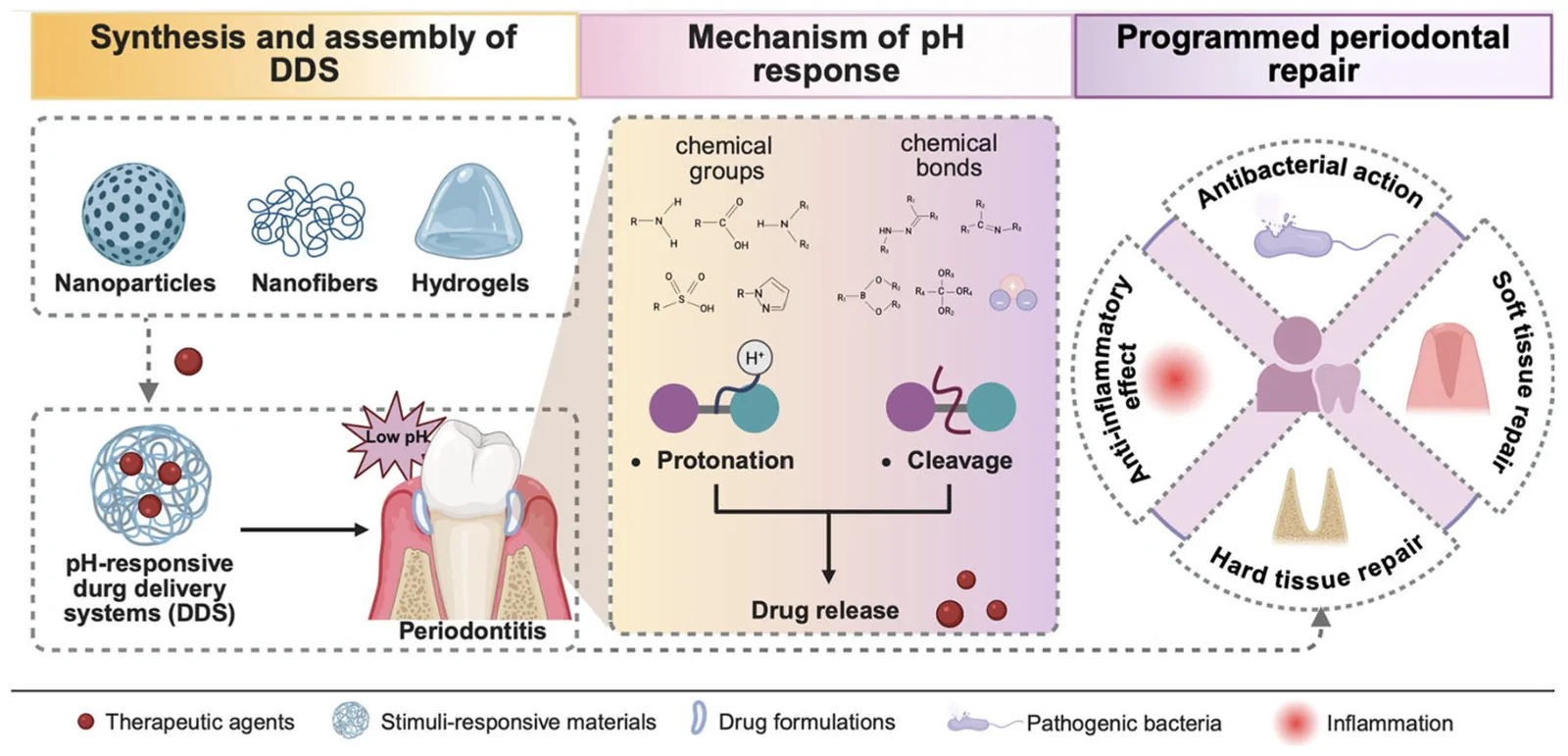
Overview of pH-responsive drug delivery systems (DDS) for periodontitis treatment. The figure summarizes three major carrier morphologies—nanoparticles (NPs), nanofibers (NFs), and hydrogels—and their respective pH-sensing mechanisms: (i) protonation/deprotonation of ionizable groups (e.g., amino groups in chitosan, carboxyl groups in carboxymethyl cellulose) leading to solubility or swelling changes; (ii) acid-labile bond cleavage (e.g., hydrazone, imine, boronate ester, and metal–ion coordination bonds) triggering carrier disassembly or drug release. The pH-responsive chemical mechanisms depicted—protonation-induced swelling and acid-catalyzed bond cleavage—are well-established physicochemical principles validated across numerous experimental systems, representative examples of which are discussed in Section 2.2.1. Reproduced with permission from ref. [13], Copyright 2025. Taylor & Francis.

Material design strategies for achieving pH-responsive sequential control are primarily based on two core mechanisms: (i) protonation or deprotonation of functional groups (e.g., amino groups in chitosan, carboxyl groups in carboxymethyl cellulose [56]), which induce alterations in solubility, swelling, or conformational changes in the material; and (ii) cleavage of pH-sensitive chemical bonds [13] (e.g., hydrazone, imine, boronate ester, and metal-ion coordination bonds). In terms of material morphology, the major forms can be categorized into nanoparticles, nanofibers, and hydrogels (Figure 5), providing a diverse toolbox for the programmed sequential therapy of periodontitis.

(1) pH-Responsive Nanoparticles (NPs): Nanoparticles have been widely employed in pH-responsive delivery owing to their high specific surface area and ease of functionalization [57]. Early research efforts primarily focused on leveraging pH responsiveness to achieve targeted delivery of antimicrobial agents, thereby enhancing anti-infective efficacy. For instance, Hu et al. [58] developed quaternized chitosan-modified, pH-activatable liposomes for doxycycline delivery, which exhibited enhanced antibacterial activity under acidic conditions. Lin et al. [59] prepared PLGA/chitosan nanospheres co-loaded with antimicrobial drugs that underwent rapid drug release at pH 5.5, significantly alleviating periodontal inflammation. Li et al. [60] loaded dexamethasone into ZIF-8 nanoparticles, which degraded under acidic conditions to release both Zn^2+^ and the drug, exerting synergistic antibacterial and anti-inflammatory effects. Beyond such single-agent antimicrobial strategies that act directly on the periodontal pocket microenvironment, the application of pH-responsive systems has been extended to the cellular level, achieving periodontal regeneration by repairing stem cell dysfunction. Zhai et al. [61] designed a multifunctional nanorepairer system (METP NPs) based on mesoporous silica nanoparticles, which—strictly speaking—represents a pH/esterase-responsive parallel dual-action platform rather than a sequentially controlled-release system in the temporal sense. It is included here because it illustrates an important conceptual extension of pH-responsive design: from controlling the order of therapeutic events to enabling coordinated multi-target intervention within a single intracellular compartment. In this system, after endocytic uptake into the acidic, esterase-rich lysosomes (pH ≈ 5.0) of MSCs, the ester bonds linking the PEG corona are cleaved, detaching the outer layer and simultaneously achieving two outcomes—exposure of the underlying EGTA chelator for mitochondrial calcium capture, and removal of the steric barrier limiting siRNA diffusion from the mesopores. Concurrently, acidic pH triggers protonation of amino groups on the MSN scaffold, weakening the electrostatic retention of siRNA by the permanently cationic trimethylammonium (TMA) anchors within the pores, with cumulative siRNA release reaching approximately 70% over 24 h. The activated EGTA captures excess Ca^2+^ around mitochondria, protecting healthy mitochondria from calcium-overload-induced deterioration, while the released siRNA targets β-catenin to inhibit the Wnt/β-catenin pathway, restoring mitophagy to clear already damaged mitochondria. These two therapeutic actions proceed in parallel—both triggered by the same lysosomal environment—and therefore the system does not embody a true temporal sequence. Nevertheless, its significance for this review lies in demonstrating that pH responsiveness can be harnessed to coordinate mechanistically distinct therapeutic modules (ion chelation and gene silencing) within a single nanoparticle platform, achieving functional synergy that complements the temporal control achieved by the sequentially releasing systems discussed in Section 2.2.3 and Section 2.2.4. In a rat periodontitis model, micro-CT analysis at 28 days confirmed that METP/siβ-catenin NP treatment significantly reduced alveolar bone loss compared with untreated controls.

In parallel with the organic and metal–organic framework-based nanocarriers discussed above, inorganic two-dimensional nanomaterials—particularly layered double hydroxides (LDHs)—have recently emerged as a versatile class of pH-responsive delivery platforms for regenerative medicine. As comprehensively reviewed by Luo et al., LDHs feature a “sandwich-like” layered structure of positively charged metal hydroxide layers with exchangeable interlayer anions, which confers several properties directly relevant to temporally controlled release [62]: (i) pH-triggered hydrolytic degradation under acidic conditions, enabling microenvironment-responsive drug release analogous to the ZIF-8 system described above, but with the added advantage of tunable degradation kinetics through compositional adjustment of the metal cation ratio; (ii) ion-exchange-mediated sustained release under physiological phosphate conditions, providing a second, complementary release mode; and (iii) exceptional compositional tunability—LDHs can incorporate a broad spectrum of biologically active metal cations (e.g., Mg^2+^ for osteogenesis, Zn^2+^ for antibacterial activity, Cu^2+^ and Co^2+^ for angiogenesis) within a single carrier, enabling intrinsic multi-functionality without additional drug loading. Although LDH-based delivery systems have demonstrated osteogenic, angiogenic, and immunomodulatory efficacy across bone, cartilage, and skin regeneration models, their application in periodontal sequential release remains unexplored, representing a significant opportunity for future investigation.

(2) pH-Responsive Nanofibers (NFs): Compared with the pH-responsive nanoparticles discussed above, the application of electrospun nanofibers in sequential controlled release for periodontal therapy remains in its infancy. Nevertheless, electrospun nanofibrous membranes exhibit distinct advantages for local periodontal drug delivery, including high specific surface area, high porosity, and tunable structural features [63]. Researchers have already successfully constructed pH-responsive nanofiber systems for the local treatment of periodontitis. Di Cristo et al. [64] fabricated quercetin (QUE)-loaded poly(lactic acid) (PLA) nanofibrous membranes. In an acidic environment at pH 4.8, the membrane served as a drug reservoir that continuously released quercetin, effectively inhibiting bacterial biofilm formation while exerting antioxidant and anti-inflammatory effects on gingival fibroblasts. Vidal-Romero et al. [65] utilized the enteric material cellulose acetate phthalate (CAP) to prepare chlorhexidine (CHX)-loaded nanofibers; the phthalate groups ionized under acidic conditions, enabling pH-triggered rapid drug release. However, the aforementioned studies have largely focused on the pH-responsive release of a single drug and have yet to address the sequential/temporal control of multiple drugs. Notably, in the field of wound healing, Guo et al. have attempted to construct pH-responsive core–shell nanofibers that rapidly release an analgesic from the shell while providing sustained release of an anti-inflammatory agent from the core, thereby achieving sequential release. Although this design principle offers valuable guidance for temporal control using pH-responsive nanofibers in the periodontal field, similar studies in periodontics remain conspicuously absent. In summary, while pH-responsive nanofibers have shown potential for local periodontal drug delivery, their extension to multi-drug sequential release systems remains an area that warrants further investigation.

(3) pH-Responsive Hydrogels: Hydrogels, with their three-dimensional hydrophilic network structure capable of accommodating substantial amounts of water and drugs, represent an excellent local delivery platform [66]. In oral medicine, hydrogels have been extensively investigated for the controlled release of antibacterial, anti-inflammatory, and osteogenesis-promoting agents [67]. Li et al. [68] designed an injectable, photo-crosslinkable hydrogel based on dynamic boronate ester covalent bonds, tailored to the dual pathological features of the diabetic periodontitis microenvironment—low pH and high reactive oxygen species (ROS) levels. This hydrogel was constructed by compositing phenylboronic acid-modified gelatin (Gel-PBA), PVA, and GelMA, with the active peptide Ac2-26 (the bioactive fragment of annexin A1) and the osteogenic growth peptide (OGP) partitioned into distinct loading domains: Ac2-26 was linked to the Gel-PBA network through pH/ROS-sensitive boronate ester bonds, whereas OGP was encapsulated within the insensitive GelMA framework. Under the low-pH stimulation of the diabetic periodontitis microenvironment, the boronate ester bonds were preferentially cleaved, triggering the rapid release of Ac2-26. This peptide suppressed macrophage pyroptosis via the ANXA1/NLRP3/caspase-1/GSDMD pathway and eliminated excess ROS, thereby creating an anti-inflammatory microenvironment conducive to tissue regeneration. Meanwhile, OGP was continuously and slowly released from the GelMA framework, promoting osteogenic differentiation and mineralization after inflammation had subsided. In vitro release profiles clearly demonstrated that Ac2-26 achieved over 70% release within 24 h in an acidic, H_2_O_2_-containing environment, whereas OGP displayed a sustained, steady release profile extending over several weeks. In a diabetic rat tooth extraction socket defect model, this system realized a sequential treatment regimen—anti-inflammatory pyroptosis suppression first, followed by bone regeneration—resulting in an approximately 2.5-fold increase in bone volume fraction (BV/TV).

Zhao et al. [69] designed a dual-immunomodulatory bone scaffold (DIBS) that exploits the dynamic pH evolution of the inflammatory microenvironment as the programming logic to precisely drive multistage sequential repair, aiming for a cascaded therapeutic goal through chemical design: anti-inflammation first, followed by enhanced immune programming, and ultimately osteogenesis. In the early inflammatory phase, the oxidized xyloglucan (OXG) in the outer hydrogel layer of the scaffold exerted a pH-independent, structural anti-inflammatory effect, directly guiding infiltrating macrophages from the pro-inflammatory M1 phenotype toward the reparative M2 phenotype. In vitro experiments confirmed that a significant decrease in CD86 and an increase in CD206 could be observed as early as day 3 of co-culture, with a dominant M2 phenotype fully established by day 7. ELISA further verified that the secretion of pro-inflammatory cytokines TNF-α and IL-6 was suppressed, whereas the anti-inflammatory cytokine IL-10 and the pro-repair factor TGF-β were significantly upregulated. Under the subsequent inflammatory low-pH conditions, the Schiff base bonds and metal coordination bonds in the Sr-PA nanoparticles within the hydrogel were responsively cleaved, accelerating the release of Sr^2+^ and protocatechualdehyde (PA). In vitro release experiments confirmed that the cumulative release of Sr^2+^ and PA under acidic conditions over 20 days reached 1.8-fold and 1.25-fold that under neutral conditions, respectively, and the release peak (days 4–16) closely coincided with the in vivo stem cell recruitment window (within 14 days post-injury). The released active factors programmed the recruited MSCs by activating the CCL2/CCR2 signaling axis to enhance their immunomodulatory functions. Single-cell RNA sequencing revealed that in the DIBS group, an MSC subpopulation highly expressing immunomodulatory genes such as *CCL2*, *CCL7*, and *CXCL1* emerged, and this subpopulation was positioned at the beginning of the pseudotime developmental trajectory, suggesting its role in establishing the immunoregenerative microenvironment. As inflammation resolved and the microenvironment pH returned to neutral, the cleavage rates of the Schiff base bonds and coordination bonds significantly slowed; the DIBS mass loss over 20 days under neutral conditions was only about 15%, far lower than the 25–30% observed under acidic conditions. The hydrogel degradation decelerated, and the scaffold function smoothly transitioned to the mineralization stage dominated by the 3D-printed HA framework, which provided mechanical support and a source of calcium and phosphate ions. In a rat calvarial defect model, DIBS achieved 23.97% new bone volume fraction (BV/TV) at 12 weeks and induced the formation of abundant type H vessels (CD31^+^Emcn^+^), confirming the synergistic regenerative effect of the immune–vascular–osteogenic cascade. Compared with the approach of Li et al., which realized sequential release of two factors by exploiting the differences in sensitive bonds within a single hydrogel network, the design logic of DIBS reflects a higher level of environmental adaptability. It not only utilizes a pH decrease to trigger the rapid release of Sr^2+^ and PA for enhanced MSC immune programming, but also, upon inflammation resolution and pH returning to neutral, automatically reduces the release rate through the markedly slowed cleavage of Schiff base bonds and coordination bonds, enabling the scaffold function to smoothly transition to HA-mediated mineralization. This responsive mechanism transforms the complete dynamic evolution of the pathological microenvironment—from acidic to neutral—into a full closed-loop switch in the release program: from activation to termination, from immunomodulation to osteogenesis promotion. Furthermore, DIBS extends the dimension of sequential regulation from factor release to the level of cellular behavior: by activating the CCL2/CCR2 pathway, it endows host MSCs with an immunomodulatory phenotype, enabling them to actively secrete chemokines that reciprocally reinforce M2 macrophage polarization and maintenance, thereby establishing a self-amplifying regenerative positive-feedback loop between macrophages and MSCs.

While the aforementioned systems have been specifically developed for periodontal applications, the broader field of dental tissue engineering has witnessed substantial progress in multifunctional hydrogel scaffold design that may inform future periodontal strategies. Guo et al. recently provided a comprehensive review of hydrogel-based scaffolds for dental pulp regeneration, summarizing the classification of natural, synthetic, and hybrid hydrogels, their crosslinking strategies, and the integration of antibacterial, angiogenic, neurogenic, and immunomodulatory functions within a single hydrogel platform [70]. Two advances highlighted in their review are of direct relevance to periodontal sequential release: (i) the development of hydrogel microspheres that enable minimally invasive injectable delivery while providing a biomimetic cellular microenvironment with sustained drug release profiles, and (ii) the emergence of 3D-bioprinted hydrogel constructs with spatially programmable architectures and patient-specific geometries. Both strategies represent design principles that could be adapted to expand the temporal and spatial control capabilities of periodontal hydrogels beyond pH responsiveness alone.

In summary, pH-responsive DDS achieve targeted and on-demand drug release by skillfully exploiting the pathological features of periodontitis. Future development will focus on: (i) constructing multi-responsive systems (e.g., pH/enzyme and pH/ROS dual-responsive systems) to address the complexity of the microenvironment; and (ii) achieving programmed repair—namely, integrating multiple functions such as antibacterial, anti-inflammatory, and osteogenesis-promoting activities within a single delivery system through sophisticated material design, while precisely controlling the sequence in which these functions are executed, thereby faithfully mimicking and guiding the natural temporal course of periodontal regeneration [13]. Although challenges remain in clinical translation, pH-responsive sequential release strategies represent one approach for developing advanced materials for periodontal regeneration.

#### 2.2.2. Enzyme-Responsive Systems

In the pathological microenvironment of periodontitis, the expression levels of various enzymes are specifically elevated [71]. Sorsa et al. [72] systematically summarized the expression patterns of the 23 members of the matrix metalloproteinase (MMP) family in periodontitis, among which MMP-8 and MMP-9 are upregulated by 5- to 6-fold in active periodontitis, while the concurrent decrease in tissue inhibitors of metalloproteinases (TIMPs) leads to an enzyme–inhibitor imbalance; MMP-12 is also found to be highly expressed in patients with chronic periodontitis. A large-scale meta-analysis further confirmed that salivary MMP-8 levels in periodontitis patients are markedly higher than those in healthy controls (SMD = 2.71) [73]. These host-derived MMPs constitute one of the principal classes of enzymes in the periodontitis microenvironment. In addition to host-derived MMPs, proteases secreted by pathogenic bacteria are also highly expressed in the periodontitis microenvironment [74]. Gingipains, secreted by *Porphyromonas gingivalis*, including arginine-specific gingipains (RgpA/RgpB) and lysine-specific gingipain (Kgp), are key virulence factors of this bacterium [75,76] and persist at sites of inflammation [74]. Dentilisin, a surface serine protease secreted by *Treponema denticola*, has also been found to be associated with elevated aMMP-8 levels [77]. These specifically elevated enzymes provide ideal endogenous triggers for the design of smart responsive drug delivery systems [78]. The core principle of enzyme-responsive systems lies in coupling the drug carrier with substrates that can be recognized by these specific enzymes (such as specific peptide sequences), thereby enabling precise and on-demand drug release [79].

The previously discussed MMP/pH dual-responsive core–shell microneedle [44] aptly illustrates this strategy: the highly expressed MMP-9 in the periodontitis microenvironment specifically cleaves the MMP-sensitive peptide sequences pre-engineered into the shell hydrogel, thereby initiating programmed shell degradation. This results in the rapid release of GA-MOF within 48 h, swiftly remodeling the anti-inflammatory microenvironment. Once the shell is nearly completely degraded, the MSC-Exo encapsulated within the core begins sustained release, achieving a synergistic combination of immunomodulation and bone regeneration. This enzyme-based sequential controlled-release strategy offers several potential advantages. First, targeting host enzymes directly links the response to the severity of inflammation rather than the presence of specific pathogens, thus affording broader applicability. Second, the shell degradation rate correlates positively with MMP concentration, allowing release kinetics to adapt dynamically to disease activity. Third, the enzyme-responsive module can be integrated with other responsive modules to increase system robustness in the complex periodontal microenvironment.

It should be noted, however, that although enzyme-responsive signals theoretically provide an ideal trigger switch for sequential controlled release, reports of single-enzyme-triggered, multistage sequential release systems in the periodontitis field remain limited. Most existing studies integrate an enzyme-responsive module with other signals such as pH [44], forming multi-responsive systems to cope with the complexity of the periodontal microenvironment. This suggests that single enzyme-responsive systems may face practical challenges in vivo, such as large fluctuations in enzyme concentration [11], insufficient substrate specificity [80], and difficulties in precisely programming degradation kinetics. Future work may focus on developing more stable and specific single-enzyme-responsive platforms, and on exploring multi-enzyme signal logic-gated systems for sequential controlled release.

#### 2.2.3. Reactive Oxygen Species (ROS)-Responsive Systems

As a typical inflammatory disease, periodontitis is characterized by excessive accumulation of reactive oxygen species (ROS) in its microenvironment. ROS (such as H_2_O_2_ and •OH) are primarily released in large quantities by infiltrating immune cells, particularly neutrophils, during the respiratory burst. While eliminating pathogens, excessive ROS also exacerbate tissue damage and destroy host cells, thereby hindering the healing process [81]. Therefore, exploiting the abnormally elevated ROS levels in the periodontal pocket as a trigger signal to construct intelligent ROS-responsive drug delivery systems [10] offers a precise approach to achieving a sequential therapeutic strategy—clearing oxidative stress first, and then initiating tissue repair.

A systematic review published by Yao et al. indicated that the core of designing ROS-responsive materials lies in using functional polymers containing ROS-sensitive chemical bonds, with their response mechanisms primarily categorized into two classes: solubility transition and chemical bond cleavage [82].

(1) Materials based on solubility transition: Under the action of ROS, the hydrophobic moieties of such materials are oxidized into hydrophilic groups, leading to fundamental changes in the physical properties of the entire material. Examples include (i) Poly(propylene sulfide) (PPS): the hydrophobic sulfide (–S–) can be oxidized to hydrophilic sulfoxide (–SO–) or sulfone (–SO_2_–), causing dissociation of the nanocarrier. (ii) Selenium-containing polymers: owing to the lower bond energy of the C–Se bond, their response is faster than that of sulfides; the hydrophobic selenide (–Se–) can be oxidized to hydrophilic selenoxide (–SeO–). (iii) Tellurium-containing polymers: these exhibit even higher ROS sensitivity than selenium-containing polymers.

(2) Materials based on chemical bond cleavage: In these materials, ROS-sensitive bonds are directly cleaved upon oxidation, leading to polymer degradation. Examples include (i) Polythioketal (PTK): the thioketal bond is highly sensitive to ROS and can be cleaved to generate ketones and thiols; PTK is widely used in tissue engineering scaffolds. (ii) Arylboronic acid/boronate esters: these bonds exhibit high specificity toward H_2_O_2_ and can react rapidly to yield phenols and boric acid, representing important tools for achieving precise drug release. (iii) Polyproline: its oxidative degradation kinetics are relatively slow, making it suitable for scaffold materials that require long-term structural maintenance.

Yao et al. [82] also systematically summarized the chemical structures, oxidation processes, and sensitivities of several key ROS-responsive materials (Table 1), providing a critical theoretical foundation and a material selection guide for designing sequential release systems tailored to the different stages of periodontitis and varying ROS concentrations [82].

Li et al. [83] reported an injectable smart hydrogel responsive to both arginine-gingipain A (RgpA) and ROS. The core design of this system lies in the use of an arginine-rich peptide (R8) as a dual-responsive element—capable of being specifically cleaved by RgpA (trigger threshold: 1 nM) and reacting with ROS (H_2_O_2_ threshold: 10^−5^ M) to generate nitric oxide (NO). RgpA, a virulence factor secreted by *Porphyromonas gingivalis*, dominates the early stage of disease; its triggered release of tannic acid (TA) and arginine exerted a rapid antibacterial effect, achieving approximately 23% inhibition at 12 h and 41% at 24 h. In the presence of both H_2_O_2_ and RgpA, the inhibition rate reached approximately 60% after 24 h, and NO was released exclusively under ROS stimulation, providing targeted anti-inflammatory action. In contrast, ROS serve as persistent signals of the inflammatory response, and their triggered release of NO focuses on anti-inflammatory and immunomodulatory actions. Although the release events triggered by the two signals overlap temporally, the therapeutic logic of this system still embodies a sequential strategy—antibacterial action first, followed by anti-inflammatory modulation—based on the natural chronological order of their appearance during the pathological process. In a mouse periodontitis model, the NHRT hydrogel group demonstrated significantly preserved alveolar bone compared with the untreated periodontitis group at both 7 and 14 days post-treatment.

Hydrogel systems based on dual ROS/pH responsiveness can precisely align with the dynamic evolution of the periodontitis microenvironment, thereby enabling the targeted and sequential release of anti-inflammatory/antioxidant factors followed by osteogenic factors. Qiu et al. [84] designed an injectable multifunctional hydrogel (HTF@HA) based on a dynamic boronate ester crosslinking network. This system uses phenylboronic acid-modified hyaluronic acid, tannic acid (TA), and Pluronic F127 as the backbone, composited with hydroxyapatite (HA), and co-loaded with concentrated growth factors (CGF) and a low dose of BMP-2. Under the conditions of high ROS and acidic pH in the periodontitis microenvironment, the dynamic boronate ester bonds are preferentially hydrolyzed, and the hydrogel network responsively undergoes accelerated degradation, allowing rapid release of TA. This quickly scavenges excessive ROS, inhibits the NF-κB pathway, rapidly exerts anti-inflammatory and antioxidant effects, and induces macrophage polarization toward the M2 phenotype, thereby creating a favorable immune microenvironment for tissue regeneration. The release kinetics of CGF and BMP-2 are markedly different from that of TA: CGF’s inherent three-dimensional fibrin network structure endows it with natural sustained-release properties, while HA adsorbs BMP-2 via calcium–phosphate interactions and achieves sustained, slow release through ion exchange, with BMP-2 cumulative release reaching 79.36 ± 0.86% by day 18. Consequently, during the early inflammatory phase, the rapid release of TA predominates, quickly controlling oxidative stress and inflammation; as the healing phase progresses and inflammation subsides, hydrogel degradation slows, TA release diminishes, and the sustained release of CGF and BMP-2 becomes dominant, continuously promoting osteogenic differentiation and angiogenesis. In a rat periodontal defect model, the HTF@HA/CGF/BMP-2 system at a low BMP-2 dose of 50 μg/L achieved bone regeneration outcomes—as assessed by micro-CT BV/TV and histological analysis at weeks 4 and 8—comparable to those of the clinical-standard high-dose BMP-2 group (500 μg/L, *p* > 0.05), representing a 10-fold reduction in the required BMP-2 dosage without compromising efficacy. This sequential, differential release strategy—rapid anti-inflammation early, followed by sustained osteogenesis promotion later—enables a temporal transition between therapeutic functions while simultaneously addressing the safety and cost concerns associated with supra-physiological growth factor dosing.

By targeting the core pathological link of oxidative stress in periodontitis, ROS-responsive systems offer a versatile approach to sequential controlled release [85]. Future development will emphasize: (i) the design of safer and more efficient ROS-sensitive materials; (ii) the construction of multi-responsive, synergistic intelligent systems that precisely align with the dynamic healing process of periodontitis; and (iii) deeper exploration of the mechanisms by which these systems regulate cell fate, to facilitate the temporal transition from antioxidant defense to pro-regenerative functions.

#### 2.2.4. Exogenous Physical Stimuli-Combined Sequential Controlled-Release Systems

Exogenous physical stimuli—most notably near-infrared (NIR) light—can serve as external trigger switches to activate or accelerate the release of therapeutic functions at a designated time point, thereby enabling precisely programmed sequential therapy. Unlike the endogenous ROS-responsive systems discussed in Section 2.2.3, which rely on pathologically elevated ROS levels in the periodontitis microenvironment, the systems described here employ an external energy input to generate the triggering signal de novo, offering the clinician temporal control over the initiation of the therapeutic cascade. Two representative NIR-triggered strategies illustrate distinct approaches to achieving sequential antibacterial-to-immunomodulatory therapy.

In a single-agent, concentration-dependent strategy, Qiu et al. [86] designed a NIR-responsive erythrocyte membrane-coated system (Hemin@ER-IR808). Upon NIR irradiation at 0.5 W/cm^2^ for 30 s, the surface-loaded photosensitizer IR808 generates H_2_O_2_, which directly damages the periodontal biofilm while simultaneously acting as a chemical switch to open the erythrocyte membrane, rapidly releasing a high concentration of hemin. The system remained stable without premature leakage for 5 days under non-irradiated conditions, confirming its on-demand release fidelity. High-concentration hemin induces ferroptosis-like stress in intracellular bacteria and promotes macrophage polarization toward the M1 phenotype through enhanced glycolysis, efficiently eliminating both intracellular and extracellular pathogens at the early stage of infection. As the erythrocyte membrane and hydrogel carrier slowly degrade, the hemin concentration declines, and low-concentration hemin activates the Nrf2/HO-1 and GPX4/SLC7A11 pathways, protecting host cells from ferroptosis and inducing macrophage polarization toward the M2 phenotype to exert antioxidant and anti-inflammatory effects, ultimately promoting alveolar bone and periodontal fiber regeneration. In a rat periodontitis model, CFU counts confirmed significant reduction in local bacterial burden at 2 weeks post-treatment, and micro-CT analysis demonstrated inhibited alveolar bone resorption at both 2 and 4 weeks, with maintained M2-polarized immunomodulation and tissue repair effects even after treatment cessation.

In a complementary bilayer spatial-partitioning strategy, Ma et al. [87] constructed a bilayer injectable small intestinal submucosa (SIS) hydrogel (IP/C@SIS), with an outer layer of SIS at 10 mg/mL loaded with ICG@PCF-1 (IP) and an inner layer of SIS at 15 mg/mL loaded with CeO_2_ nanoparticles. The indocyanine green (ICG) is a NIR photosensitizer with dual photodynamic and photothermal functions; PCF-1, a hydrogen-bonded organic framework, encapsulates ICG in situ within its pores through π–π stacking and hydrogen bonding, markedly improving ICG photostability and ROS generation efficiency. Under 808 nm NIR irradiation, the outer IP converts surrounding oxygen into highly oxidative singlet oxygen, efficiently destroying the periodontal biofilm, while the photothermal effect raises the local temperature to approximately 43 °C, synergistically enhancing antibacterial action and accelerating outer hydrogel degradation. The cumulative IP release over 240 h reached approximately 41.8% without NIR but exceeded 70% with NIR irradiation. The inner SIS layer—formulated at a higher concentration (15 mg/mL) with a denser crosslinked structure and physically enveloped by the outer layer—exhibited markedly delayed CeO_2_ release during the early stage, with a cumulative release of less than 8% within 13 days; the outer layer was nearly fully degraded by day 10, after which CeO_2_ was continuously released, scavenging residual ROS through its superoxide dismutase- and catalase-like activities, inhibiting M1 macrophage polarization, promoting M2 polarization, and acting synergistically with the abundant type I/III collagen, VEGF, FGF-2, and other growth factors in the SIS matrix to achieve alveolar bone and periodontal fiber regeneration. In a rat ligature-induced periodontitis model, the IP/C@SIS+ group (with NIR irradiation at 0, 24, 48, 96, and 144 h post-injection) demonstrated the shortest cementoenamel junction-to-alveolar bone crest (CEJ–ABC) distance and the highest bone volume/collagen volume (BV/CV) ratio among all treatment groups at 42 days post-surgery; near-complete eradication of both Gram-positive and Gram-negative bacteria was achieved in the irradiated groups.

These two systems embody complementary design philosophies for NIR-triggered sequential therapy. The Qiu et al. [86] approach achieves functional transition through concentration-dependent biological switching of a single agent (hemin), minimizing the number of active components but requiring precise control over local drug concentration. The Ma et al. [87] approach achieves temporal separation through the spatial partitioning of two distinct functional agents (IP and CeO_2_) across a bilayer structure with differential degradation kinetics, offering independent tunability of each phase at the cost of greater fabrication complexity. Both strategies demonstrate that exogenous NIR irradiation can serve as a clinically convenient, externally controllable trigger for programmed periodontal therapy—transforming a single physical input into a temporally resolved cascade of antibacterial, anti-inflammatory, and regenerative outputs.

Beyond NIR-based approaches, other exogenous physical stimuli—including ultrasound and magnetic fields—have also been explored as trigger switches for sequential control, although their application in periodontal therapy remains at an early stage. Future development in this area will likely emphasize: (i) the design of photosensitizers with higher ROS quantum yields and absorption in the NIR-II window (1000–1700 nm) for deeper tissue penetration; (ii) portable, patient-friendly light sources suitable for intraoral application; and (iii) the integration of exogenous triggers with endogenous microenvironment-responsive modules to construct dual-lock sequential release systems with enhanced spatiotemporal precision.

#### 2.2.5. Comparative Assessment

A cross-strategy comparison of the four stimuli-responsive categories reveals fundamental trade-offs among trigger specificity, release autonomy, and clinical practicability that distinguish these environment-interactive systems from the structurally programmed diffusion-barrier platforms discussed in Section 2.1.

(1) Evidence strength: All stimuli-responsive systems surveyed in this section have been evaluated exclusively in small-animal models—predominantly rat ligature-induced periodontitis or calvarial defect models, with one system validated in a mouse periodontitis model [83]—and none has progressed to large-animal (canine, porcine) testing. Among the four subcategories, pH-responsive hydrogels carry the most extensively replicated in vivo evidence base, with at least three independent systems demonstrating significant alveolar bone preservation or regeneration in rat models: the Ac2-26/OGP dual-responsive hydrogel achieved an approximately 2.5-fold increase in bone volume fraction (BV/TV) in a diabetic rat tooth extraction socket model [68], the dual-immunomodulatory bone scaffold (DIBS) attained 23.97% new bone BV/TV at 12 weeks in a rat calvarial defect model [69], and the HTF@HA/CGF/BMP-2 hydrogel produced bone regeneration at a 10-fold-reduced BMP-2 dose (50 vs. 500 μg/L, *p* > 0.05) in a rat periodontal defect model [84]. ROS-responsive systems have two independent in vivo validations at the rodent level: the RgpA/ROS dual-responsive NHRT hydrogel significantly preserved alveolar bone in a mouse ligature-induced periodontitis model at both 7 and 14 days post-treatment [83], and the HTF@HA hydrogel (classified under ROS/pH dual-responsive systems) demonstrated osteogenic efficacy in a rat periodontal defect model [84]. NIR-triggered systems have two independent in vivo validations: the Hemin@ER-IR808 system confirmed inhibition of alveolar bone resorption at 2 and 4 weeks in a rat periodontitis model [86], and the IP/C@SIS bilayer hydrogel demonstrated periodontal tissue regeneration [87]. Enzyme-responsive systems, considered as a standalone triggering strategy, present a distinctive evidence profile: two dual-responsive systems incorporating enzyme-cleavable modules—the MMP/pH dual-responsive core–shell microneedle [44] in a rat periodontitis model, and the RgpA/ROS dual-responsive NHRT hydrogel [83] in a mouse periodontitis model—have demonstrated in vivo therapeutic efficacy. However, in both cases the enzyme-responsive module is coupled with a second, complementary trigger (pH or ROS), and a pure single-enzyme-triggered sequential release platform—i.e., one whose release program is governed solely by enzyme concentration—has yet to be demonstrated in any periodontal model. This pattern—enzyme sensing consistently appearing in dual-responsive rather than single-trigger configurations—suggests that enzyme concentration alone may be insufficiently robust as a standalone release trigger in the complex periodontal microenvironment, where enzyme levels fluctuate substantially across disease stages and among individuals [79]. As noted in Section 2.1.4, direct cross-study comparison of quantitative bone morphometric outcomes is methodologically unsound; comparisons are best anchored to evidence tier and independent replication count.

(2) Trigger specificity and release autonomy: The four subcategories occupy distinct positions along a specificity–practicality spectrum that has important translational implications. Enzyme-responsive systems offer the highest biological specificity: MMP-8 and MMP-9 are upregulated 5- to 6-fold specifically in active periodontitis [72], while RgpA is uniquely derived from the keystone periodontal pathogen *P. gingivalis* [83], making these triggers intimately linked to disease etiology. This specificity, however, is counterbalanced by substantial inter-patient and intra-patient variability in enzyme concentrations [79], which complicates the design of a release schedule that performs predictably across individuals. pH-responsive systems benefit from a well-characterized pathological pH gradient (pH ~5.5 in inflamed pockets vs. ~7.4 in healthy tissue [53]) and a mature chemical toolbox (boronate esters, Schiff bases, metal-coordination bonds), but pocket pH is influenced by dietary intake, smoking, and other confounding factors that introduce noise into the trigger signal. ROS-responsive systems directly target a core pathological mechanism (oxidative stress-driven tissue damage) and can achieve dual therapeutic effects—ROS scavenging and triggered drug release—within a single material; however, the response thresholds of current ROS-sensitive materials (typically 10^−5^ to 10^−6^ M H_2_O_2_ in vitro [83]) have not been systematically calibrated to clinically measured periodontal ROS concentrations, creating uncertainty about in vivo activation fidelity. Exogenous physical stimuli (NIR) occupy a unique position on this spectrum: they offer clinician-controlled, on-demand triggering entirely independent of variable endogenous conditions, enabling the most precisely programmable release timing. This advantage is offset by the need for external equipment (laser source, intraoral light-delivery device), a treatment schedule tied to clinical visits rather than continuous autonomous action, and NIR tissue penetration depth limitations (~3–5 mm at 808 nm) that may constrain applicability to deep intrabony defects.

(3) Translational readiness: From a clinical translation perspective, pH-responsive hydrogels currently lead across several practical dimensions: injectable formulations compatible with existing syringe-based clinical workflows, reliance on endogenous triggers requiring no external equipment, and the largest replicated in vivo evidence base. ROS-responsive systems share the first two advantages and offer the added mechanistic benefit of simultaneously addressing oxidative stress pathology—a feature of particular relevance to periodontitis associated with systemic conditions such as diabetes. However, their translational development lags behind pH-responsive systems in independent replication. Enzyme-responsive systems, despite their superior biological specificity, face distinct translational barriers: patient-specific enzyme activity profiling would likely be necessary to predict release kinetics with confidence, and the repertoire of enzyme-cleavable linkers with established clinical-grade safety profiles remains limited [79]. The consistent co-occurrence of enzyme-responsive modules with a second trigger (pH or ROS) in all existing in vivo-validated systems further suggests that clinical translation of enzyme-responsive technology may depend on multi-responsive integration rather than standalone deployment. NIR-triggered systems encounter unique challenges including regulatory classification as combined drug–device products, the requirement for intraoral light-delivery devices with patient-acceptable form factors, and the current absence of standardized irradiation protocols for periodontal indications. A notable cross-cutting observation is that many of the most effective systems in this section—the MMP/pH dual-responsive microneedle [44], the RgpA/ROS dual-responsive hydrogel [83], and the ROS/pH dual-responsive HTF@HA hydrogel [84]—integrate two trigger modalities rather than relying on a single stimulus. This convergence toward multi-responsive architectures suggests that the optimal strategy for addressing the complexity of the periodontal microenvironment may lie not in selecting a single superior trigger, but in engineering systems that combine the continuous surveillance capability of endogenous sensing (pH, enzyme, ROS) with orthogonal validation across multiple pathological signals, thereby increasing the robustness of release activation. Across all four subcategories, the most critical translational gap remains the absence of large-animal validation; until release kinetics and therapeutic efficacy are demonstrated in canine or porcine periodontal defect models, the translational potential of any stimuli-responsive strategy remains provisional.

### 2.3. Sequential Control Based on Cell/Extracellular Vesicle Systems

#### 2.3.1. Mechanism and Current Strategies

Delivery systems that exploit natural biological processes can also achieve sequential control while simultaneously offering exceptionally high biocompatibility and precision [88]. The principle of such systems is to use cell membranes or extracellular vesicles as carriers, whose inherent inflammatory tropism and cell-homing capabilities enable targeted delivery [89]. Through engineering modifications, these carriers can be made to respond to microenvironmental changes upon reaching the lesion site and release their contents in a programmed manner [90]. Cui et al. [91] developed an engineered M2 macrophage-derived exosome system loaded with melatonin (Mel@M2-exos). This system first capitalizes on the intrinsic inflammation-targeting ability of M2 macrophage-derived exosomes (M2-exos) to actively recognize and be preferentially taken up in large quantities by M1 macrophages at the inflammatory site. In vivo fluorescence imaging tracing experiments showed that DiD-labeled M2-exos accumulated at the periodontal inflammatory site as early as 8 h after injection, reaching peak enrichment at 24 h before gradually fading. In vitro experiments also confirmed that LPS-activated inflammatory macrophages internalized M2-exos at significantly higher levels than non-activated cells. This efficient targeted uptake ensured that the subsequent immunomodulatory program could be directly initiated within the M1 macrophages residing at the core of the lesion.

Following uptake by M1 macrophages, M2-exos rapidly initiated immune reprogramming of the host cells, driving their transition from a pro-inflammatory to an anti-inflammatory phenotype. At the protein level, M2 macrophage markers such as CD163 and Arg1 could be directly transferred through membrane fusion and other mechanisms to convey phenotypic signals; at the genetic level, nucleic acid molecules carried by the exosomes could enter the host cells and initiate the phenotypic conversion program at the transcriptional level. This reprogramming process ultimately suppressed the pro-inflammatory activity of M1 macrophages, as evidenced by decreased expression of CD86 and iNOS and increased expression of CD206 and Arg1, thereby creating a favorable immune microenvironment for subsequent tissue regeneration.

On this basis, as the M2-exos degraded intracellularly, the loaded melatonin was continuously released and acted on periodontal ligament cells (hPDLCs). This delayed sustained release avoided the pro-inflammatory and cytotoxic side effects associated with high-concentration melatonin. It significantly suppressed the LPS-induced overexpression of endoplasmic reticulum stress markers such as GRP78 and CHOP, modulated key signaling molecules in the UPR pathway including PERK, ATF6, and IRE1, and consequently downregulated CHOP, inhibited caspase-3 activation, and upregulated Bcl-2, thereby breaking the vicious cycle of inflammation-driven ER stress and apoptosis. Once the cells were freed from the threat of apoptosis, the previously suppressed expression of osteogenic and cementogenic genes and proteins—such as *ALP*, *COL1*, and *Runx2*—was reactivated. When loaded into an injectable GelMA hydrogel, Mel@M2-exos significantly inhibited alveolar bone resorption in a rat periodontitis model, achieving a closed-loop sequential therapy from immunomodulation to tissue regeneration.

#### 2.3.2. Manufacturing and Translational Challenges

Despite the unique biological advantages of EV- and cell-based delivery platforms, their path toward clinical translation in periodontal therapy is encumbered by several interconnected manufacturing and regulatory hurdles that are distinct from those facing the synthetic material systems discussed in Section 2.1 and Section 2.2.

(1) Scalability: The production of therapeutic EVs at clinically relevant quantities remains a formidable bottleneck. Current isolation protocols—predominantly ultracentrifugation, size-exclusion chromatography, and tangential flow filtration (TFF)—are hindered by inherently low recovery rates and cumulative sample loss across processing steps [92]. At the industrial scale, EV yields rarely exceed 10^13^ particles per liter of conditioned medium, a figure that remains substantially below the projected dose requirements for many therapeutic indications [93]. For periodontal applications, the required dose per defect is likely lower than that for systemic administration, yet the combination of donor cell expansion under good manufacturing practice (GMP) conditions and the intrinsic inefficiency of EV biogenesis means that even a single clinical-grade batch demands bioreactor-scale culture and downstream processing pipelines that have only recently begun to be established [92,94]. Three-dimensional culture platforms offer a promising path forward: microcarrier-based stirred-tank systems achieve approximately a 5.7-fold increase in EV yield over conventional 2D monolayer culture, and when paired with TFF-based isolation, the combined platform can boost output by up to 140-fold [94]. In a representative hollow-fiber bioreactor study, an average of 8.1 × 10^10^ particles/mL was harvested from conditioned medium in a fully closed-system configuration, thereby simultaneously enhancing yield and minimizing contamination risk [94]. For stem cell-based systems, the scalability challenge is analogous but compounded by cellular biology: primary mesenchymal stromal cells (MSCs) must be expanded through multiple passages to reach clinically meaningful cell numbers, yet each passage carries the risk of replicative senescence, phenotypic drift, and progressive loss of paracrine potency—all of which directly erode the sequential signaling that these systems are designed to deliver [95,96,97].

(2) Batch-to-batch variability: EV composition—including the repertoire of proteins, lipids, and nucleic acids that collectively determine therapeutic function—is exquisitely sensitive to the state of the parental cell. Variables such as donor age and health status, cell passage number, serum-derived EV contamination in culture media, and environmental conditions (oxygen tension, inflammatory priming, mechanical stimulation) can all produce substantial differences in EV cargo and bioactivity across production runs [93,95]. The MISEV2018 guidelines and their 2023 update (MISEV2023) from the International Society for Extracellular Vesicles recommend rigorous characterization across at least three orthogonal categories—particle concentration and size distribution, transmembrane or cytosolic protein markers (e.g., CD9, CD63, CD81, TSG101), and biochemical composition or functional activity—and MISEV2023 further requires that the limit of detection for each quantitative method be explicitly reported [98,99]. Despite these consensus recommendations, the field lacks certified reference materials and universally accepted potency standards that would enable inter-batch comparability at the level required for regulatory approval—a gap underscored by evidence that even harmonized multicenter manufacturing protocols reduce, but do not eliminate, donor- and tissue-source-dependent variability [92,95]. A parallel problem exists for MSC products: cells derived from different tissue sources (bone marrow, dental pulp, periodontal ligament, adipose tissue) exhibit distinct transcriptional signatures and secretory profiles, and even cells from the same tissue source can vary substantially among donors, introducing fundamental uncertainty into the reproducibility of cell-based sequential therapies [95,97].

(3) Storage stability: Unlike synthetic drug delivery systems that can often be lyophilized and stored at ambient temperature, EV- and cell-based products are inherently labile. A systematic review of 50 studies on EV storage concluded that −80 °C is the optimal long-term storage temperature, whereas storage at 4 °C is viable for only approximately one week and multiple freeze–thaw cycles cause significant reductions in particle concentration, RNA cargo integrity, and bioactivity [100]. Although lyophilization with disaccharide cryoprotectants has shown considerable promise—a trehalose/sucrose mixture at 4% each (*w*/*v*) preserved EV concentration, size, morphology, and protein/RNA content for up to 30 days at room temperature by inhibiting buffer salt crystallization through amorphous glass formation—therapeutic activity began to decline after 7 days, indicating that residual moisture control and functional preservation remain incompletely solved [101]. Moreover, the optimal lyoprotectant formulation varies with EV source and cargo, and universally applicable protocols have yet to be validated across diverse EV product types [100,101]. Current best practice therefore requires storage at −80 °C, imposing a cold-chain burden that complicates clinical deployment outside of specialized academic centers. For live cell products, the stability challenge is more severe: cryopreserved MSCs typically require immediate thawing and administration at the point of care, as post-thaw viability and immunomodulatory potency decline within hours; fresh cell preparations must be used within a narrow time window, precluding conventional inventory management and distribution models [97]. These constraints are particularly problematic for periodontal therapy, where clinical adoption depends on products that can be stored in a dental office setting and applied during a routine outpatient procedure.

(4) Regulatory considerations: The regulatory classification of EV- and cell-based therapies critically shapes the development pathway and evidentiary requirements for clinical translation. Engineered EVs—especially those that are surface-modified, loaded with exogenous nucleic acids or small-molecule drugs, or derived from genetically manipulated producer cells—may be classified as biological products, advanced therapy medicinal products (ATMPs, in the European Union), or combination products (drug–device or drug–biologic), depending on the nature and extent of modification [102,103]. A 2025 analysis of the global regulatory landscape noted that no regulatory agency worldwide has yet issued EV-specific drug evaluation guidelines, and the classification criteria for EV products vary significantly across the U.S. Food and Drug Administration (FDA), European Medicines Agency (EMA), and China’s National Medical Products Administration (NMPA), creating uncertainty for developers seeking to design pivotal trials acceptable to multiple jurisdictions [102]. Mesenchymal stromal cell products face a regulatory landscape that is comparatively more mature—the FDA’s Regenerative Medicine Advanced Therapy (RMAT) designation and the EMA’s ATMP framework provide defined pathways—but as of 2024, only 11 MSC-based products have received marketing authorization worldwide, predominantly in Asia (South Korea, Japan, India, Iran), with the first U.S. MSC approval (remestemcel-L for pediatric graft-versus-host disease) occurring only in December 2024 [97]. The requirement for demonstration of potency, consistency, and long-term safety in large-animal models before initiating human trials translates into development timelines and costs that far exceed those for synthetic material-based drug delivery systems. To date, no EV- or stem cell-based product has received regulatory approval specifically for periodontal regeneration, although the clinical trial pipeline for MSC-based therapies in related craniofacial applications—including alveolar ridge augmentation and sinus floor elevation—provides an emerging roadmap for the evidentiary standards that will likely be applied to periodontal indications [97,102].

Collectively, these manufacturing and regulatory challenges do not diminish the therapeutic promise of EV- and cell-based sequential delivery platforms, but they underscore that the pathway from preclinical proof-of-concept to clinical adoption will require parallel advances in bioprocess engineering, quality-by-design manufacturing frameworks, and regulatory science—domains that have not historically been the focus of periodontal biomaterials research but are now indispensable for translation.

### 2.4. Temporal Functional Synergy Based on Asymmetric Structural Design

By designing materials with inherently asymmetric structures or functional domains, researchers have enabled them to exert synergistic functions at different time points post-implantation, thereby realizing a sequential therapeutic logic that emphasizes barrier or anti-inflammatory functions in the early phase and transitions to pro-regenerative functions in the later phase [104]. An early exploration of this concept was the guided bone regeneration (GBR) membrane with a bilayered structure [105]. The design philosophy was to deconstruct complex physiological requirements in the spatial dimension: the dense side faces the soft tissue and acts as an inert physical barrier, while the porous side faces the bone defect to support cell migration and nutrient exchange. Such designs achieved dual functions in a single membrane and have achieved widespread clinical success. From a strict sequential control perspective, however, the functions of the two sides are typically initiated simultaneously upon implantation and operate continuously thereafter, representing essentially a static spatial partitioning of functions rather than an active relay logic in the temporal dimension [106].

In recent years, asymmetric structural design has been extended from the macroscopic physical level to the microscopic molecular release level, achieving more intelligent sequential responses. Chen et al. [107] constructed a polyphenol-based intelligent oral barrier membrane (PBMC) by employing a layer-by-layer self-assembly strategy on a collagen barrier membrane. This membrane not only retained the traditional dual-sided structure of collagen membranes—a smooth side to block epithelial cell migration and a rough side to promote cell attachment—but also introduced a ROS-responsive intelligent coating, achieving molecular-level temporal functional synergy of inflammation modulation and antibacterial action first, followed by osteogenesis promotion. In early-stage periodontitis, high ROS levels triggered the rapid, controllable release of minocycline from the membrane, exerting an early anti-infective effect. Concurrently, the natural polyphenols loaded in the membrane continuously scavenged excess ROS, reversing the pro-inflammatory microenvironment and creating favorable conditions for the subsequent migration and osteogenic differentiation of human periodontal ligament stem cells, thereby realizing an automated sequential repair process from debridement to tissue regeneration. In a rat periodontal bone defect model with postoperative *Porphyromonas gingivalis* inoculation to simulate implant-associated infection, the PBMC-treated groups demonstrated a significantly reduced CEJ–ABC distance relative to the untreated and collagen-only controls at both 4 and 6 weeks post-surgery; micro-CT analysis further revealed elevated bone volume fraction (BV/TV) and trabecular number (Tb.N) and decreased trabecular separation (Tb.Sp) in the PBMC-treated groups. Histological examination at 6 weeks confirmed substantial new alveolar bone formation accompanied by neovascularization and a robust collagen matrix, and immunohistochemical staining demonstrated significantly increased expression of the early osteogenic marker RUNX2 and the late-stage marker osteocalcin (OCN) at the defect margins (*p* < 0.0001 versus control and collagen groups).

**Figure 6:**
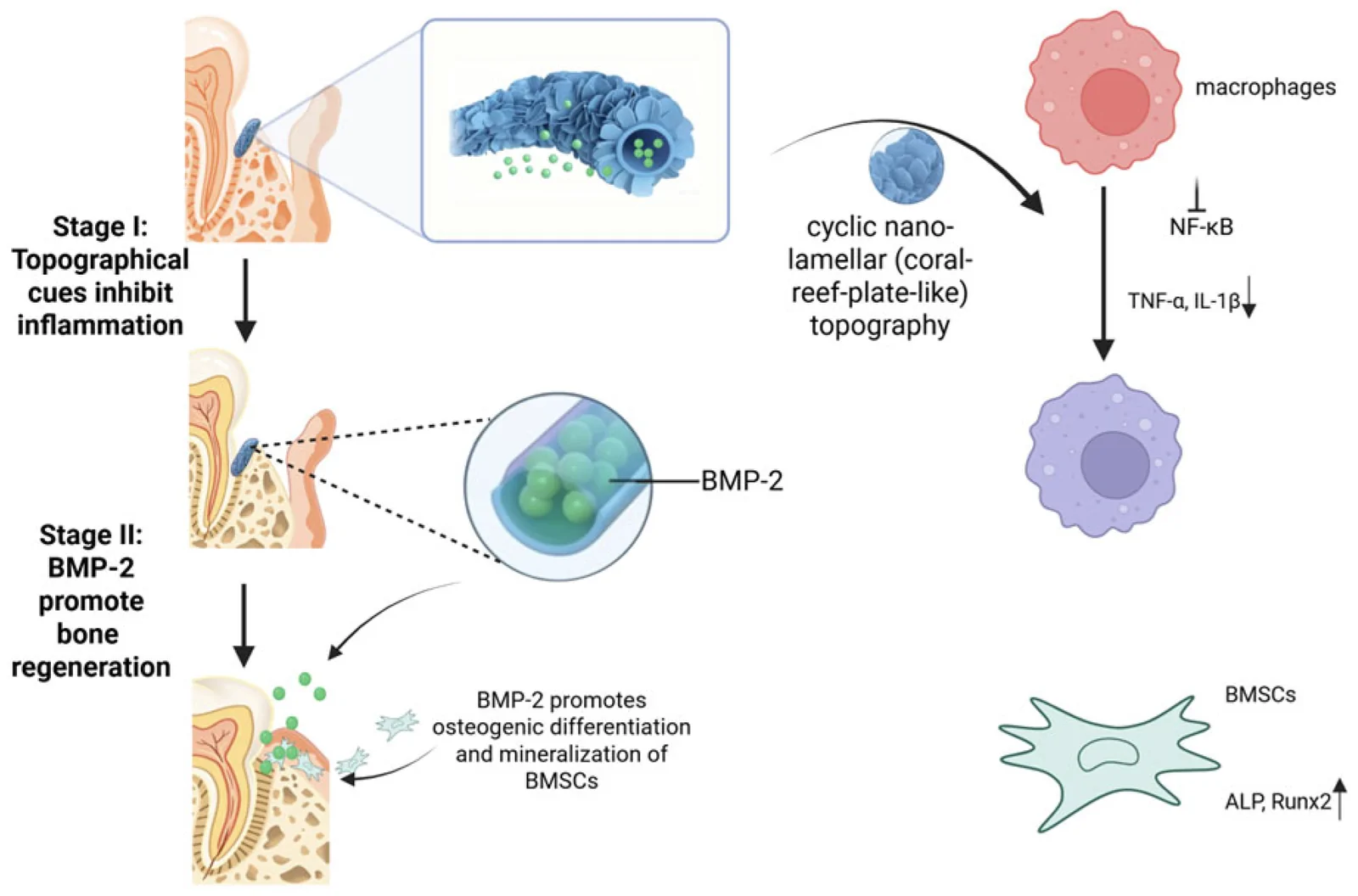
Hierarchically structured nanofibrous scaffold (TS@BMP-2) achieving spatiotemporal modulation of the osteoimmune microenvironment through dual topographical and biochemical cues. A core–shell nanofiber is fabricated by coaxial electrospinning: BMP-2 is loaded in the poly(vinyl alcohol) (PVA) core, and cyclic nano-lamellar (coral-reef-plate-like) topography is constructed on the polycaprolactone (PCL) shell via surface-directed epitaxial crystallization. This architecture embeds a structure-inherent temporal logic: (Early phase) The nano-topographical cues on the fiber surface serve as intrinsic physical signals that attenuate macrophage inflammation via NF-κB pathway inhibition, establishing a regeneration-conducive osteoimmune microenvironment without requiring exogenous biochemical triggers. (Late phase) Concurrently, the ultra-thin PCL shell (~45 nm) permits sustained diffusional release of BMP-2 from the PVA core—achieving zero-order kinetics over the initial 9 days and ~82% cumulative release by day 24—promoting osteogenic differentiation and mineralization of bone mesenchymal stem cells after inflammation is controlled. The depicted mechanisms—topography-driven macrophage modulation, NF-κB pathway inhibition, and core–shell sustained BMP-2 release via diffusion—were experimentally demonstrated in vitro (macrophage co-culture, BMSC differentiation assays, ELISA release profiling) and validated in vivo in a mouse periodontitis model (immunofluorescence for macrophage markers, micro-CT, histology) by He et al. [108]. (The figure was created with BioRender.com).

An alternative strategy involves integrating intrinsic physical signals with releasable biochemical signals through multi-level asymmetric structures, creating a sequentially programmed therapeutic procedure governed primarily by the structural logic of the material itself rather than exogenous environmental triggers. He et al. [108] designed a hierarchically structured nanofibrous scaffold (TS@BMP-2), whose core innovation resides in constructing dual topographical and biochemical cues within a core–shell architecture (Figure 6). First, a core–shell structure was formed by coaxial electrospinning, with BMP-2 loaded into the poly(vinyl alcohol) (PVA) core. Subsequently, cyclic nanolamellar (coral-reef-plate-like) nanotopographical features were constructed on the polycaprolactone (PCL) shell through surface-directed epitaxial crystallization. This exquisite multi-level structure achieved sequential spatiotemporal orchestration: the early commands were dominated by the topographical shell, where the nanotopography on the fiber surface served as an intrinsic physical signal that directly regulated macrophages, modulating them toward an anti-inflammatory phenotype by inhibiting the NF-κB pathway. This shaped an osteoimmune microenvironment conducive to regeneration during the initial stage of healing and completed the critical step of immunomodulation. The late commands were then executed by the biochemical core, where the core–shell structure ensured the sustained-release effect of BMP-2, such that its peak action occurred after inflammation was controlled, thereby promoting the osteogenic differentiation and mineralization of bone mesenchymal stem cells to achieve the repair goal of tissue regeneration. In a mouse ligature-induced periodontitis model, the TS@BMP-2 scaffold outperformed all control groups (plain PCL shell, PS; PS with BMP-2, PS@BMP-2; and topographical shell without BMP-2, TS) at both 1 and 4 weeks post-implantation: micro-CT analysis showed that the TS@BMP-2 group exhibited the highest BV/TV and trabecular number (Tb.N), the lowest trabecular separation (Tb.Sp), and the shortest CEJ–ABC distance, indicating the least alveolar bone resorption among all groups. Immunohistochemical staining confirmed the lowest expression of the pro-inflammatory cytokine IL-1β and the fewest TRAP-positive osteoclasts in the TS@BMP-2 group, while osteogenic activity at 4 weeks was the highest. Notably, the TS group (topographical cues alone) also significantly suppressed inflammation and bone resorption relative to the control and PS groups, confirming that the nanotopography exerts a stand-alone therapeutic effect independent of BMP-2 release. This study reveals an optimized design for temporal functional synergy: through the multi-level asymmetric structure of the material, physical and biochemical signals are spatiotemporally orchestrated to actively guide tissue regeneration along the natural healing program. This structure-inherent temporal logic offers an alternative to environmentally passive response strategies and may inform future material design for periodontal regeneration.

### 2.5. Cross-Strategy Comparative Assessment: From Design Philosophy to Clinical Translation

The four temporal control strategies surveyed in Section 2.1, Section 2.2, Section 2.3 and Section 2.4—diffusion barrier-based systems, stimuli-responsive materials, cell/extracellular vesicle (EV)-based delivery, and asymmetric structural design—each embody a distinct design philosophy and occupy a different position along the translational trajectory. This section provides a cross-strategy comparison along four dimensions: therapeutic logic, evidence strength, manufacturing feasibility, and translational readiness. The aim is not to declare any single strategy superior, but to identify the conditions under which each approach is most likely to succeed and to highlight the gaps that currently separate preclinical proof-of-concept from clinical adoption in periodontal therapy.

#### 2.5.1. Therapeutic Logic Spectrum: From Engineering Pre-Programming to Bio-Inspired Delivery

The four strategies can be arranged along a continuum defined by the source and sophistication of their temporal control logic. At one end lie diffusion-barrier systems (Section 2.1), whose release sequences are pre-programmed into the material architecture: the order and timing of drug release are governed by the spatial arrangement of compartments, differential degradation kinetics, and passive diffusion distances. Once implanted, the program executes without feedback from the local biological environment. Asymmetric structural designs (Section 2.4) extend this pre-programmed logic into the spatial dimension—directing different therapeutic functions to different tissue compartments—but, as discussed below, the systems reviewed in that section often couple structural asymmetry with stimuli-responsive elements, placing them at the interface between purely structural and environment-sensing strategies [107,108]. At the midpoint, stimuli-responsive systems (Section 2.2) introduce a layer of environmental awareness—they remain quiescent until pathological cues (acidic pH, elevated MMPs, excess ROS, or an exogenous NIR trigger) initiate therapeutic release. This sensing capability represents an advance beyond purely structural control, yet the relationship remains unidirectional: the system responds to the environment but does not actively shape it beyond the intended therapeutic action. At the far end, cell- and EV-based systems (Section 2.3) share a bio-derived delivery logic: unlike purely synthetic carriers, their ability to recognize and interact with target tissues is governed by molecular fingerprints inherited from parental cells [88,91], enabling a degree of biological targeting that synthetic systems cannot replicate. Collectively, they represent a distinct class of bio-inspired delivery platforms that prioritize biological recognition over engineering pre-programming, albeit through distinct operational mechanisms. The core trade-off across this continuum is controllability versus biological fidelity: strategies positioned toward the structural end offer greater predictability and manufacturing simplicity, while those toward the biological end provide superior targeting specificity and biocompatibility—at the cost of less engineering control over the timing, dose, and composition of the delivered payload.

#### 2.5.2. Evidence Strength Across Strategies

A critical comparison of the preclinical evidence base reveals a stark and instructive asymmetry. Among all systems surveyed in Section 2.1, Section 2.2, Section 2.3 and Section 2.4, only one—the core–shell micelle-in-nanofiber membrane developed by Liu et al. (Section 2.1.1)—has been evaluated in a large-animal periodontal model (Beagle dog, Class II furcation defect), providing micro-CT, histomorphometric, and clinical attachment level data [26]. The remaining systems, spanning all four strategy categories, have been tested exclusively in small-animal models—predominantly rat or mouse periodontitis induced by ligature or LPS injection, or rodent calvarial defect models—with the exception of the CAPP multilayer film, which has not progressed beyond in vitro characterization.

This evidence distribution warrants careful interpretation. Small-animal periodontal defect models, while indispensable for mechanistic proof-of-concept, differ from human periodontitis in several physiologically significant respects: faster bone turnover rates, a distinct subgingival microbiota, substantially smaller defect dimensions (typically 10- to 50-fold smaller than human intrabony defects), and a healing trajectory that is less dependent on mechanical stability and microbial control than is the case in the human oral cavity [109]. Positive outcomes in rodent models should therefore be regarded as encouraging but insufficient, on their own, to establish translational credibility.

Within the EV-based category (Section 2.3.1), the current review discusses one principal system—Mel@M2-exos (Cui et al. [91])—that has demonstrated in vivo efficacy in a rat periodontitis model.

A methodological caveat, echoed from Section 2.1.4, applies across all strategy categories: direct numerical comparison of bone morphometric outcomes (BV/TV, CEJ–ABC distance, new bone area) between studies is invalid owing to differences in animal species, defect geometry, observation time points, micro-CT acquisition parameters, and region-of-interest definitions. Cross-strategy comparison is therefore anchored to evidence tier—large animal > small animal disease model > small animal ectopic/surgical defect > in vitro only—rather than to numerical efficacy values.

#### 2.5.3. Design Complexity and Manufacturing Feasibility

A clear gradient in design complexity and manufacturing feasibility parallels the evidence-strength hierarchy established in Section 2.5.2: the strategy with the strongest preclinical evidence (diffusion barrier systems, Section 2.1) is also the most manufacturing-tractable. Coaxial electrospinning, emulsion-based microsphere fabrication, and micromolding for microneedle arrays are established industrial processes with defined quality control parameters; their design complexity resides in geometry and material selection rather than in dynamic chemical functionality, which simplifies process validation and batch-to-batch reproducibility. Stimuli-responsive systems (Section 2.2) introduce an additional layer of complexity through multi-step fabrication—each responsive moiety must be independently synthesized, incorporated, and characterized—and their release performance is inherently context-dependent on the local biochemical milieu (see also Section 3.2.1). EV-based systems (Section 2.3) face the most severe manufacturing hurdles, as detailed in Section 2.3.2. The DELIVER translational framework proposed by Joyce et al. (2024) provides a useful comparative lens: diffusion barrier systems satisfy a larger fraction of the DELIVER criteria at the current stage of development than do stimuli-responsive or EV-based systems, primarily because their manufacturing workflows are more reproducible and their critical quality attributes are more completely defined [110]. A detailed discussion of manufacturing scalability, batch-to-batch variability, and regulatory considerations across all strategy categories is deferred to Section 3.2.6.

#### 2.5.4. Translational Readiness and Recommended Pathway

Drawing together the preceding analyses of evidence strength, manufacturing feasibility, and design complexity, we offer the following assessment of translational readiness. These projections represent the authors’ judgment based on the evidence surveyed in this review; they are not derivable directly from any single cited study and should be read as informed estimates subject to revision as new data emerge.

Diffusion barrier systems—particularly core–shell nanofiber membranes [26]—are, in our assessment, the nearest-term translational candidates among the four strategies. Their combination of large-animal efficacy data, mature manufacturing processes, and a comparatively straightforward regulatory pathway (as Class II/III medical devices rather than biologics, depending on the drug-loading configuration and jurisdictional classification) supports a tentative estimate of 3–7 years to first-in-human clinical evaluation. This timeline is contingent on completion of GLP toxicology studies, large-animal periodontal defect healing studies with clinically relevant outcome measures, and demonstration of superior efficacy over existing clinical standards (e.g., enamel matrix derivative or collagen membranes alone).

pH-responsive hydrogels (Section 2.2.1) represent, in our judgment, the most promising mid-term strategy, with a tentative translational window of 5–10 years. Their advantages—injectability, compatibility with minimally invasive periodontal surgery, and the ability to couple drug release to the natural resolution of inflammation—address genuine clinical needs. Two representative systems, the Ac2-26/OGP-loaded boronate ester hydrogel [68] and the dual-immunomodulatory bone scaffold (DIBS) [69], have demonstrated that pH-programmed sequential therapy can substantially enhance bone regeneration in rodent models (2.5-fold BV/TV increase and 23.97% new bone volume, respectively). The principal barrier to clinical translation is the need to demonstrate that pH-responsive release kinetics are sufficiently reproducible across the heterogeneous biochemical landscape of human periodontitis to support predictable clinical outcomes. The clinical validation of antimicrobial photodynamic therapy delivered via transgingival near-infrared irradiation [111]—a randomized controlled trial in 40 patients—provides a proof-of-principle that externally triggered periodontal therapies can be successfully implemented in the dental operatory and suggests a regulatory pathway for devices that combine physical energy delivery with local drug release.

EV-based systems face a longer translational horizon, which we estimate at 10 or more years given the combined weight of manufacturing scalability, batch-to-batch reproducibility, storage stability, and regulatory classification uncertainties detailed in Section 2.3.2. This projection does not diminish the therapeutic promise of EV-based delivery; rather, it reflects the reality that the bioprocess engineering and regulatory science infrastructure required for clinical-grade EV manufacturing is still under construction. The clinical trial pipeline for MSC-based therapies in craniofacial applications—including alveolar ridge augmentation and sinus floor elevation—provides an emerging evidentiary template (see Section 2.3.2), but no EV-based product has yet reached Phase II/III trials for a periodontal indication.

Asymmetric structural design, as discussed in Section 2.5.1, is best understood as a cross-cutting architectural principle that can enhance the spatial precision of release when integrated with any of the other three strategies. Its translational trajectory is therefore coupled to the strategy with which it is paired.

The recommended development pathway is not serial—pursue Strategy A, then B, then C—but parallel and stratified by risk. Core–shell nanofiber membranes, as the lowest-risk, highest-evidence strategy, merit prioritization for near-term clinical translation efforts. pH-responsive hydrogels should be developed in parallel, with particular attention to the characterization of inter-patient variability in intra-pocket pH dynamics and its impact on release reproducibility. EV-based systems require, first and foremost, convergence on standardized manufacturing and characterization frameworks—efforts that are underway in the broader EV community and that will benefit periodontal applications once mature. The asymmetric structural design principle should be incorporated into each of these strategies wherever spatial release precision adds therapeutic value, rather than pursued as an isolated development track.

A comparative summary of all systems discussed in Section 2.1, Section 2.2, Section 2.3 and Section 2.4, including their release mechanisms, therapeutic agents, key preclinical evidence, advantages, limitations, and clinical status, is provided in Table 2.

## 3. Advantages and Challenges of Sequential Controlled-Release Systems

### 3.1. Core Advantages

Approximating the physiological healing rhythm. Sequential controlled-release systems can dynamically adapt to the distinct biological demands of the inflammatory, proliferative, and remodeling phases during periodontal healing. By following a release logic of anti-inflammation or antibacterial action first and osteogenesis promotion later, these systems effectively circumvent the temporal antagonism among different bioactive factors, thereby potentially enhancing regenerative efficiency and quality [112].

Synergistic enhancement of multiple therapeutic effects. By optimizing the sequence of action and the spatiotemporal distribution of different factors, these systems can produce synergistic therapeutic effects. For instance, scavenging reactive oxygen species (ROS) or modulating macrophage polarization during the early inflammatory phase creates a favorable immune microenvironment for subsequent stem cell-mediated bone regeneration [78,86].

Reducing side effects through temporal control. Sequential control prevents growth factors from being prematurely exposed to ROS or proteases during the inflammatory phase, which would lead to their inactivation, and it also averts the risks of ectopic ossification or pathological mineralization that can arise when high concentrations of growth factors are released at the wrong time [25].

### 3.2. Major Challenges

#### 3.2.1. Precise Prediction of Release Kinetics and Individualized Regulation

The efficacy of sequential controlled-release systems rests on whether their release kinetics can be precisely executed according to the preprogrammed schedule in vivo—a requirement undermined by three interrelated sources of uncertainty. First, standard in vitro release tests cannot replicate the dynamic flow of gingival crevicular fluid, the complex biomolecular milieu, or the metabolic activities of the microbiota within the periodontal pocket; these factors can markedly alter material degradation rate, swelling behavior, and drug diffusion coefficient, leading to severe deviations between in vitro predictions and in vivo performance [113]. Second, even for well-designed smart materials, response threshold, speed, and reversibility within the dynamically changing inflammatory microenvironment are difficult to quantify precisely. The triggered release of pH- or enzyme-responsive materials, for example, is heavily dependent on the instantaneous local concentration of signaling molecules, which can fluctuate substantially among patients and at different time points within the same patient [114]. Third, and most critically, substantial interindividual variability—in periodontal pocket anatomy, gingival crevicular fluid flow rate and composition, host immune status, microbial community structure, and oral hygiene habits—renders the periodontal microenvironment highly heterogeneous [115], such that any release system predicated on a single or average set of parameters will struggle to meet the demands of individualized therapy. Collectively, these three sources of uncertainty—incomplete environmental mimicry in preclinical testing, context-dependent material responsiveness, and patient-to-patient heterogeneity—constitute the core challenge that must be overcome for clinical translation in this field [116,117].

#### 3.2.2. Long-Term Biocompatibility and Safety Concerns of Carrier Materials

The long-term biocompatibility and safety of carrier materials are the cornerstone of their clinical translation; however, significant concerns and knowledge gaps persist in current research [118]. First, the potential long-term risks of various advanced materials have not been fully elucidated. Taking metal–organic frameworks (MOFs) as an example, their degradation in physiological environments may result in the sustained release of metal ions (e.g., Zn^2+^, Zr^4+^) and organic ligands. The long-term in vivo fate of these degradation products—including their metabolic pathways, potential for target organ accumulation, and clearance mechanisms—remains unclear, posing risks of unknown immune reactions, cytotoxicity, or chronic tissue injury [119]. Moreover, for many stimuli-responsive polymers, the ultimate degradation products following the response have also not been systematically evaluated for their long-term biological effects [120]. Second, there is a severe lack of systematic long-term in vivo safety evaluation systems [121]. The vast majority of studies remain limited to short-term in vitro cytocompatibility tests or animal observations spanning only a few weeks, which cannot predict the long-term behavior of materials over months or years in the human body [122]. A complete biological safety evaluation should encompass systemic toxicity, immunogenicity, carcinogenic and teratogenic potential, and the long-term stability of the interface between the material and host tissue after repair is completed. To date, preclinical safety assessment models and data that can simulate complex human microenvironments—such as chronic inflammation and dynamic mechanical loading—over extended periods are extremely scarce [121]. Finally, inter-individual variability further exacerbates the complexity of safety prediction [88]. Differences in genetic background, immune status, metabolic capacity, and periodontal microbiome among patients can lead to profoundly different biological responses to the same material [123]. Such person-specific reactions introduce uncertainty when safety conclusions derived from standard models are applied to clinical practice [124]. Therefore, the lack of reliable models and data that can predict the long-term in vivo behavior of materials and their complex interactions with the host has become a core safety bottleneck hindering the clinical translation of these intelligent systems.

#### 3.2.3. Retention and Mechanical Stability of Materials in the Dynamic Moist Environment

The realization of sequential controlled-release systems depends not only on intricate material design and release kinetics, but also on their long-term retention and structural stability within the dynamic, moist, and mechanically active microenvironment of the periodontal pocket [14]. The sustained flow of gingival crevicular fluid [125], salivary washout, masticatory forces, and tongue-and-cheek movements constitute a highly challenging physicochemical environment [67]. If a drug-loaded system dislocates, detaches, or undergoes structural collapse within a short period (e.g., within hours) after implantation, its pre-programmed sequential release procedure will completely fail, becoming merely a theoretical exercise [126]. Currently, the in vitro release models employed in most studies are usually established under static or simple fluid conditions, making it difficult to replicate the complex fluid dynamics and mechanical perturbations within the oral cavity [113]. Although some studies have introduced bioadhesive materials (e.g., chitosan, hyaluronic acid, polydopamine coatings) or physical interlocking structures (e.g., microneedles, porous scaffolds) to enhance the bonding strength between the material and the tooth root or soft tissue, the long-term adhesion durability, washout resistance, and mechanical integrity of these strategies under wet conditions have yet to be systematically validated [127]. This is especially true for patients with chronic periodontitis, where increased gingival crevicular fluid flow and inflammatory exudate further aggravate the retention challenge [123]. In addition, the mechanical stability of materials is also a key factor affecting functional persistence [128]. Under daily functional loading such as chewing and tooth brushing, brittle materials are prone to fracture, whereas flexible materials may gradually lose structural integrity due to fatigue, resulting in burst drug release or disordered release programs [129]. Therefore, future material design must pay greater attention to wet-state adhesive mechanics, anti-fatigue characteristics, and long-term shape recovery capabilities [130], and it must promote the establishment of dynamic in vitro models and mechanical testing standards that are more clinically realistic, so as to evaluate the retention performance and structural durability of the systems under simulated oral environments [15].

#### 3.2.4. Translational Bottleneck of Animal Models

Animal models serve as an indispensable bridge for evaluating the therapeutic efficacy of sequential controlled-release systems, but their inherent limitations constitute a significant bottleneck for clinical translation [5]. First, at the level of model foundation: commonly used small-animal models (e.g., rat, mouse) differ markedly from humans in terms of periodontal defect dimensions, bone regeneration rate, salivary composition, and oral microbial community, making it difficult to adequately replicate the complex anatomical structure, fluid mechanical environment, and chronic inflammatory processes of the human periodontal pocket [131]. Second, at the level of evaluation criteria: animal experiments typically focus on short-term (weeks) histological and radiographic repair outcomes, whereas human periodontal regeneration pursues long-term (years) functional stability and clinical attachment gain, resulting in a mismatch between the two evaluation systems [132]. More importantly, at the level of mechanistic validation: animal models cannot faithfully mirror human-specific immune responses, individualized healing capacity, or complex social-behavioral factors such as oral hygiene compliance, all of which profoundly influence the in vivo fate and ultimate therapeutic efficacy of sequential controlled systems [133]. Consequently, even for systems that perform excellently in animal experiments, substantial uncertainties remain regarding the in vivo predictability of their release kinetics, long-term biosafety, and functional robustness within the real human pathological microenvironment [5].

#### 3.2.5. Precise Regulation of Complex Factor Networks

Periodontal regeneration is not governed by a single signal but rather involves an intricate regulatory network comprising numerous genes, metabolites, and signaling pathways [134], posing a fundamental challenge to achieving true sequential control. The molecular regulatory network of periodontal healing is highly complex and dynamic [135]. For example, multi-omics studies have revealed an interaction network involving dozens of key genes and metabolites in periodontitis, which together finely orchestrate multiple critical pathways including cytokine signaling and purine metabolism [136]. This implies that tissue regeneration is not simply a superposition of linear commands but rather a dynamic system characterized by redundancy, feedback, and compensatory mechanisms, where intervention at any single node may trigger unpredictable network cascade effects [137]. The current design paradigm for sequential release systems primarily focuses on coordinating the release sequence of two to three key bioactive factors. This simplified strategy of regulating only a small number of factors has adequately validated the feasibility of the sequential therapeutic logic and has laid the groundwork for more refined regulation. However, there remains an orders-of-magnitude dimensional gap between this and the complex, dynamic regulatory network comprising hundreds of molecular components upon which periodontal tissue regeneration actually depends [136]. Achieving functional regeneration demands not only the release of multiple factors but also the precise mimicking of their dynamic concentration ratios, spatiotemporal distributions, and the temporal rhythm of their interactions, which places extremely high demands on the intelligent design and control logic of the materials [138]. Thus, the ultimate core challenge is to propel sequential release from the current simple paradigm of regulating a few factors toward intelligent network programming based on a systems-level regulatory logic [139]. This requires integrating the precision controlled-release capabilities of materials science with a deep understanding of network dynamics from systems biology [140], shifting the delivery system from passively executing a pre-programmed schedule to actively engaging in a dynamic dialog with the tissue microenvironment [141]. This leap represents the fundamental scientific challenge that must be overcome to realize truly functional tissue regeneration.

#### 3.2.6. Manufacturing Complexity and Scalability

Beyond the biological and physicochemical challenges discussed in Section 3.2.1, Section 3.2.2, Section 3.2.3, Section 3.2.4 and Section 3.2.5, the translation of sequential controlled-release systems faces a more fundamental hurdle: whether these architecturally elaborate constructs can be manufactured reproducibly, at scale, and under conditions that satisfy regulatory standards. Whereas Section 2.3.2 has addressed manufacturing challenges specific to cell- and extracellular vesicle-based therapies, the present section considers manufacturing barriers common to all four strategy categories.

Manufacturing complexity increases with the number of discrete components and fabrication steps. Diffusion barrier-based systems (Section 2.1)—relying on coaxial electrospinning, emulsion templating, and micromolding—employ unit operations with established industrial precedents and comparatively well-defined critical quality attributes (CQAs), including fiber diameter, shell thickness, and drug loading uniformity [142]. Stimuli-responsive systems (Section 2.2) introduce substantially greater complexity: each responsive moiety must be independently synthesized, incorporated at a controlled density, and characterized for sensitivity, selectivity, and response kinetics. In multi-step constructs—such as hydrogels combining enzyme-cleavable and stable network domains within a single matrix [68,69]—each orthogonal crosslinking step introduces an additional source of batch-to-batch variability. Herrmann et al. (2025) identified this accumulated manufacturing complexity as a persistent barrier separating laboratory demonstrations from clinical adoption [117], and the DELIVER framework proposed by Joyce et al. (2024) similarly designates manufacturing reproducibility as a core criterion for translational readiness [110].

The transition from laboratory-scale synthesis (milligrams to grams) to Good Manufacturing Practice (GMP)-compliant production (grams to kilograms) is seldom addressed in the preclinical periodontal literature. Batch processes dominant in academic laboratories—bulk nanoprecipitation, manual electrospinning—suffer from declining parameter control at larger reactor volumes, manifesting as increased polydispersity and reduced encapsulation efficiency [143]. Continuous manufacturing platforms, particularly microfluidics-based approaches that replace volumetric scale-up with parallelized numbering-up of identical small-volume reactors, offer a potential strategy for preserving the precise control achievable at the microscale while increasing throughput [144]; their application to multi-component sequential release systems, however, remains at an early stage [145]. An additional and frequently overlooked consideration is terminal sterilization: common modalities including gamma irradiation and autoclaving can alter polymer molecular weight, crosslinking density, and degradation kinetics in widely used materials such as chitosan, alginate, and Pluronic F-127, yet systematic risk-assessment frameworks for sterilizing stimuli-sensitive multi-component constructs remain underdeveloped [146]. Finally, regulatory classification uncertainty—these systems may be regulated as medical devices, drug products, combination products, or biologics depending on composition and primary mechanism of action—introduces ambiguity into manufacturing planning from the earliest stages of translational development.

## 4. Summary and Outlook

Sequential controlled-release systems represent a developing approach for periodontal regeneration, reflecting a shift in treatment strategy from passive drug release to temporally regulated delivery. Future research should focus on the following aspects:

(1) Advancing toward multi-responsive and logic-gated systems. The dual-responsive systems discussed in Section 2.2—integrating pH with ROS, MMP with pH, or RgpA with ROS sensing—represent the current state of the art in periodontal sequential release. The next conceptual advance lies in embedding Boolean logic operations (AND, OR, NOT) directly into material design, such that therapeutic release is gated not by a single environmental cue but by specific combinations thereof. This paradigm was recently demonstrated in a landmark study by Gharios et al., who engineered hydrogel networks in which protein cargo release was governed by orthogonal protease-sensitive linkers arranged in series (OR gate: either protease suffices) or in parallel (AND gate: both proteases required), successfully implementing all 17 possible YES/OR/AND logic operations from three distinct protease inputs [147]. A comprehensive framework proposed by Rehorst and Kros formalized the design dimensions of logic-gated nanocarriers—including logic architecture, molecular implementation strategy, and the distinction between digital-like (protein-based) versus analog (chemically responsive) activation thresholds [148]. For periodontal applications, an illustrative AND-gate design would require simultaneous low pH and elevated MMP-9—both characteristic of active periodontitis but not of resolved lesions—to trigger drug release, thereby confining therapeutic action both spatially and temporally to sites of active disease. DNA-based logic gates and aptamer-functionalized hydrogels responsive to multiple small-molecule or protein inputs represent additional molecular toolkits that could, in principle, be adapted for intraoral use. Critical hurdles include the limited stability of DNA nanostructures in nuclease-rich oral fluids, the need for reversible rather than one-shot logic responses to accommodate the fluctuating inflammatory milieu, and the integration of logic-gated release with clinically practical delivery form factors such as injectable hydrogels or periodontal fibers. Addressing these challenges will require interdisciplinary collaboration among DNA nanotechnologists, polymer chemists, and periodontal clinicians.

(2) Deeply integrating biotechnology and immunomodulation. The cell- and extracellular vesicle-based systems reviewed in Section 2.3 have established the therapeutic principle that biological carriers can simultaneously target inflammatory sites and reprogram the local immune milieu. The next frontier is the convergence of these cell-based delivery platforms with programmable gene-editing and nucleic acid therapeutics. CRISPR-Cas systems are being actively explored for periodontal applications along two distinct axes: species-specific depletion of keystone pathogens such as *Porphyromonas gingivalis* without disrupting the commensal oral microbiome, and host-targeted gene editing to durably modulate inflammatory signaling pathways, as comprehensively reviewed by Chopra et al. [149]. In parallel, lipid nanoparticle (LNP)-mediated mRNA and miRNA delivery is emerging as a clinically tractable strategy for periodontal tissue regeneration. Ding et al. demonstrated that miR-27 mimic delivered via LNPs promoted periodontal regeneration through coordinated osteogenesis and angiogenesis—suppressing the Wnt inhibitor SFRP1 to achieve a 43.9% reduction in the alveolar bone–cementoenamel junction distance and a six-fold increase in neovascularization in a rodent periodontitis model [150]. Complementing tissue-level repair, Yoon et al. developed piperazine-derived bisphosphonate-functionalized LNPs that bind tightly to hydroxyapatite, creating a local mRNA reservoir on bone and tooth surfaces—a strategy directly applicable to periodontal defect sites where mineralized tissue targeting is desired [151]. It must be acknowledged that these gene-based approaches share, and in some respects amplify, the manufacturing and regulatory challenges detailed in Section 2.3.2 and Section 3.2.6: vector immunogenicity, batch-to-batch variability in LNP formulation, and the regulatory complexity of classifying gene-modified therapeutics. Nevertheless, the accelerating clinical translation of mRNA-LNP platforms—catalyzed by the COVID-19 vaccine experience—provides a regulatory and manufacturing precedent that EV- and cell-based therapies have yet to establish, suggesting that nucleic acid delivery may represent a nearer-term translational path than living-cell products for periodontal sequential therapy.

(3) Embracing personalized precision medicine. The substantial interindividual variability in periodontitis severity, inflammatory microenvironment composition, and host healing capacity—documented across the systems surveyed in this review—makes it unlikely that any single fixed-sequence release program will be optimal for all patients [152]. Personalized sequential control will require two converging capabilities: real-time, chairside monitoring of disease activity to inform release parameter selection, and delivery platforms whose release kinetics can be tuned to individual patient profiles. Toward the first capability, salivary point-of-care testing has now reached a level of clinical validation that makes biomarker-guided treatment personalization conceivable: a 2024 meta-analysis of six studies confirmed that the active matrix metalloproteinase-8 (aMMP-8) chairside test achieves a pooled specificity of 0.84 for detecting periodontitis, and a recent comprehensive review by Alavi et al. catalogued the expanding repertoire of salivary biomarkers—including cytokines, microbial DNA, and exosomal miRNAs—that could, in principle, inform the selection and timing of sequential release interventions [153,154]. Toward the second capability, the emerging concept of closed-loop drug delivery—in which a biosensor continuously monitors a disease-relevant parameter and an integrated actuator adjusts drug release accordingly—has been systematically reviewed by Paci et al., who identified wearable and implantable platforms integrating real-time biosensing with automated therapeutic release as a technically feasible near-term goal [155,156]. For periodontal applications, a closed-loop system could, in concept, monitor gingival crevicular fluid pH or MMP-8 levels via an intraoral sensor and wirelessly adjust the degradation rate of an implanted sequential release hydrogel through triggered crosslink cleavage—though substantial engineering challenges in miniaturization, power management, and intraoral environmental stability remain. Realizing such closed-loop control will demand computational methods distinct from those employed for materials design: specifically, reinforcement learning or model-predictive control algorithms capable of processing time-varying biomarker signals and adaptively adjusting the crosslink cleavage rate, swelling ratio, or diffusion barrier integrity of the implanted construct in response to the patient’s evolving inflammatory status. The development and validation of such control algorithms for the biochemically noisy and interindividually variable periodontal microenvironment represents a significant research program in its own right, requiring close integration of intraoral sensor engineering, control theory, and periodontal biology.

(4) Leveraging artificial intelligence and big data-driven design. The rational design of sequential controlled-release systems requires navigating a high-dimensional parameter space encompassing material composition, scaffold architecture, drug loading distribution, and the interplay among diffusion, swelling, and degradation mechanisms—a complexity that exceeds the capacity of empirical trial-and-error optimization alone. Machine learning (ML) models, particularly artificial neural networks and ensemble methods such as XGBoost, have demonstrated superior accuracy over traditional empirical models (e.g., Higuchi, Korsmeyer–Peppas) in predicting drug release profiles from polymeric carriers, by capturing the nonlinear relationships among formulation variables, material properties, and release kinetics [157,158]. A recent comprehensive survey of ML-empowered nanoparticulate formulation design documents the successful application of these methods across diverse carrier platforms—including polymeric nanoparticles, liposomes, and micelles—for predicting particle size, encapsulation efficiency, and release behavior [159]. Beyond forward prediction, generative AI and inverse design strategies are emerging: genetic algorithms coupled with fused deposition modeling 3D printing have been employed to autonomously design multi-layer capsule geometries that produce prescribed stepwise and zero-order release profiles, demonstrating that AI can directly optimize the temporal sequence of multi-drug release without manual parameter tuning [160]. Complementing purely data-driven approaches, physics-informed neural networks (PINNs) embed governing diffusion and mass-transfer equations directly into neural network architectures, yielding mechanistically interpretable predictions of drug release kinetics while requiring substantially less training data than conventional deep learning models [161]. For periodontal sequential delivery specifically, these computational tools could, in principle, be trained on existing in vitro release datasets to predict the temporal release profiles of candidate multi-drug formulations in silico, rank competing material designs by their predicted release sequence fidelity, and identify optimal dosing intervals between sequential therapeutic phases—substantially reducing the experimental burden of formulation screening. Critical barriers to clinical implementation remain: the scarcity of standardized, high-quality drug release datasets specific to periodontal delivery systems limits model generalizability; the “black-box” nature of many ML architectures raises regulatory acceptance concerns; and the gap between in silico predictions and in vivo performance in the biochemically dynamic periodontal microenvironment has yet to be systematically evaluated. Addressing these challenges will require the establishment of FAIR-compliant drug release databases, the continued development of hybrid physics-informed models with greater interpretability, and prospective experimental validation of computationally optimized formulations in animal models of periodontitis.

(5) Strengthening clinical translational research. As the cross-strategy comparison in Section 2.5 and the manufacturing analysis in Section 3.2.6 have established, the four strategy categories span a wide gradient of translational readiness—from diffusion barrier-based systems, which combine the strongest preclinical evidence with the most mature manufacturing workflows, to EV- and cell-based systems, which offer the highest biological sophistication but face the steepest scalability and regulatory hurdles [110]. Accelerating translation across this spectrum will require three parallel efforts. First, systematic preclinical validation in large-animal models—dogs and mini-pigs, whose periodontal anatomy and healing kinetics more closely approximate those of humans than do rodent models—must become the standard rather than the exception; a 2025 review by Podembski et al. identified only 12 in vivo studies of PDLSC-based therapies across all animal models, of which just 4 employed dogs or pigs, underscoring the paucity of large-animal data even for the most clinically advanced regenerative strategies [162,163]. Second, manufacturing processes must be designed for scalability from the outset: continuous microfluidic platforms and 3D/4D printing technologies offer routes to standardized, batch-reproducible fabrication of architecturally complex sequential release constructs, as discussed in Section 3.2.6 [144]. Third, the regulatory classification ambiguity noted in Section 3.2.6—these combination products may fall under medical device, drug, or biologic frameworks depending on composition and primary mechanism of action—must be proactively addressed through early engagement with regulatory agencies, ideally during the preclinical proof-of-concept phase. Adaptive clinical trial designs capable of evaluating both the safety and the sequential therapeutic logic of these multi-component systems will be essential, as conventional parallel-arm superiority trials are poorly suited to capturing the temporal dimension of therapeutic benefit that defines this class of interventions.

In conclusion, the evidence assembled in this review supports a clear translational gradient: diffusion barrier-based systems are built on industrially mature unit operations with defined critical quality attributes and carry the strongest preclinical evidence. These systems are best positioned for near-term clinical adoption. Stimuli-responsive systems offer greater biological adaptability but must resolve the challenge of context-dependent release reproducibility, while EV- and cell-based platforms, though biologically the most sophisticated, face manufacturing and regulatory hurdles that will likely defer their clinical arrival. Across this spectrum, systematic large-animal validation, scalable manufacturing from the earliest design stage, and proactive regulatory engagement must advance in parallel with continued materials innovation. The field is now poised to move beyond proof-of-concept demonstrations toward the rigorous translational development that will determine whether these temporally engineered systems can fulfill their therapeutic promise for periodontal patients.

## Figures and Tables

**Table 1:** The oxidation of ROS-responsive polymeric materials.

ROS-Responsive Materials	Chemical Structure and Oxidation	Sensitivity	Material Format	Reaction Time
Poly(propylene sulfide)	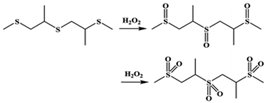	H_2_O_2_: 3.3 vol% 3-Morpholinosydnonimine (SIN-1): 1–100 mM Peroxynitrite: 1–100 μM	Polymeric micelles	24 h
Thioether	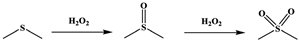	H_2_O_2_: 100 μM	Polymeric NPs	24 h
Selenium-containing polymers	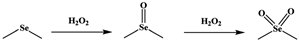	H_2_O_2_: 0.1% *v*/*v*	Polymeric aggregates	10 h
	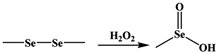	H_2_O_2_: 0.001% *v*/*v* Oxygen radicals	Polymeric micelles	6 h
Tellurium-containing polymers	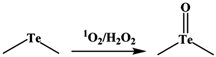	H_2_O_2_: 100 μM	Spherical aggregates	1 h
Poly(thioketal)	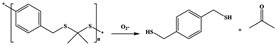	O_2_−: 0.2 mM	Polymeric NPs	4 h
	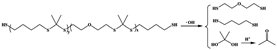	H_2_O_2_: 0.2% *v*/*v*	Polymeric scaffolds	10 days
Phenylboronic acid and ester-containing polymers	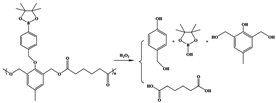	H_2_O_2_: 50 μM	Polymeric NPs	24 h
	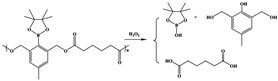	H_2_O_2_: 100 μM	Polymeric NPs	24 h
Poly(proline)	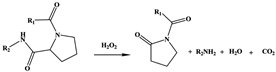	SIN-1: 1 mM H_2_O_2_: 5 mM	Polymeric scaffolds	24 h

**Table 2:** Comparative Summary of Temporally Controlled Release Systems for Periodontal Regeneration.

System Information				Release Characteristics	Key Evidence		Assessment		
Strategy Category	Representative System	Release Mechanism	Therapeutic Agent(s)	Release Time Window	Key In Vitro Data	In Vivo Model & Key Outcomes	Advantages	Limitations	Clinical Status
A. Diffusion Barrier & Degradation Kinetics Systems (Section 2.1)									
Layered film	CAPP multilayer film (Sundararaj et al. 2013 [25])	Solvent-evaporation lamination; PSA coating ensures unidirectional surface erosion; blank spacer layers prolong release intervals	Metronidazole (antibacterial), Ketoprofen (anti-inflammatory), Doxycycline (anti-resorptive), Simvastatin (osteogenic)	MTZ: 15–25 h; KTP: ~40–60 h; DOX: ~40–100 h; SIM: innermost layer, released last. Total erosion time ~160 h.	Release sequence strictly follows layer order; MTZ and KTP retain ≈100% bioactivity post-encapsulation.	No in vivo data—in vitro release characterization and bioactivity testing only.	Among the earliest explorations of the sequential release concept; four-stage temporal sequence validated in vitro.	No in vivo validation; substantial overlap between nominally sequential phases; >10 years since publication without further advancement.	In vitro proof-of-concept (lowest evidence tier)
Core–shell nanofiber	Core–shell micelle-in-nanofiber membrane (Liu X. et al. 2020 [20])	Coaxial electrospinning; SP600125 in PEG-b-PCL micelles → gelatin shell; BMP-2 in PVA core	SP600125 (JNK inhibitor, anti-inflammatory), BMP-2 (osteogenic)	Shell SP600125: ~41–47% within 4 d, >75% cumulative by 28 d. Core BMP-2: first detected at d 12, ~100 ng/mL by d 16, sustained to d 22.	SP600125 early suppression of LPS-induced IL-1β, IL-6, MMP-2, MMP-9; BMP-2 late upregulation of ALP, Runx2 (d14), OPN, OCN (d21).	Beagle dog Class II furcation defect (5 mm Ø × 2 mm): single implantation → significant inhibition of alveolar bone destruction + bone defect repair at 2 mo (micro-CT BV/TV + sequential fluorochrome labeling).	Only system with large-animal evidence; temporal separation via BMP-2 detection delay (~12 d); single-step surgical intervention.	Only two drugs; SP600125 is a broad-spectrum JNK inhibitor, not periodontium-specific.	Preclinical—large animal (highest evidence tier)
Core–shell scaffold	PGA-SF core–shell electrospun scaffold (Jiang X. et al. 2024 [21])	Coaxial electrospinning; IL-4 physically adsorbed on SF shell; TCP nanoparticles encapsulated in PGA core	IL-4 (anti-inflammatory cytokine, immunomodulation), TCP (osteoconductive)	IL-4: >70% within 3 d, sustained ≥7 d. Ca^2+^: peaks at d 7–10, detectable up to d 46.	RAW 264.7–BMSC co-culture: M2↑ (Arg-1, CD206), M1↓ (TNF-α, iNOS, IL-1β); BMSC osteogenesis↑ (ALP, ARS, RUNX2, OCN). Optimal IL-4 loading: 156.55 ng/cm^2^.	Rat critical-size calvarial defect: BV/TV↑, Tb.N↑, Tb.Sp↓; 2-wk IF: CD206^+^ M2↑, iNOS^+^ M1↓.	Dual-mode release (rapid immunomodulation + prolonged osteogenesis); exploits differential diffusion resistance of physical adsorption vs. matrix encapsulation.	Rat calvarial defect only (non-periodontitis model); physical adsorption of IL-4 may introduce batch variability.	Preclinical—small animal
Core–shell + LbL nanofiber	LbL-assembled core–shell nanofiber membrane (Cheng G. et al. 2019 [22])	Coaxial electrospinning + layer-by-layer (LbL) electrostatic self-assembly; SF/PCL shell, PVA core; CTGF deposited on fiber surface via chitosan–CTGF bilayers	CTGF (connective tissue growth factor, angiogenic/chemotactic), BMP-2 (osteogenic)	CTGF: burst d 1, maintained ~5 d, largely complete by d 10, ~90% cumulative over 40 d. BMP-2: sustained linear release >30 d, minimal initial burst.	LbL deposition dual function: CTGF reservoir + suppression of BMP-2 initial burst. Optimized formulation: SF/PCL = 1:5, 20 LbL bilayers.	Mouse calvarial defect: sequential dual-delivery → 43% improvement in bone regeneration vs. single BMP-2 (micro-CT). Fluorescent labeling confirmed CTGF signal undetectable by d 15, BMP-2 signal persisted to d 30.	Superposition of independent spatial segregation strategies (coaxial + LbL) in a single construct → expanded temporal complexity; faithfully recapitulates natural CTGF→BMP-2 healing sequence.	Multi-step fabrication; mouse calvarial defect only (non-periodontitis model).	Preclinical—small animal
Cell-carrier microsphere	CGRP-loaded porous PLGA/PDA microspheres (Luo J. et al. 2023 [29])	CGRP physically encapsulated in PLGA/PDA porous microspheres; BMSCs co-delivered; CGRP sustained release → inflammation resolution → BMSC survival → osteogenesis	CGRP (calcitonin gene-related peptide, anti-inflammatory/immunomodulatory), BMSCs (stem cells)	CGRP: 6247.4 ± 44.7 pg cumulative by d 8 (loading ~0.35 ng/mg microspheres).	CGRP inhibits ROS/NLRP3/caspase-1 pathway → ↓IL-1β; protects BMSCs from inflammation-induced apoptosis.	Mouse ligature-induced periodontitis: PM/CGRP/BMSCs BV/TV = 84.07% vs. PM/BMSCs 76.39% vs. PM/CGRP 55.83% vs. PBS 37.18%; BMD: 1003.16 vs. 807.57 mgHA/cm^3^.	Extends temporal logic from drug release to cell therapy—CGRP creates a survivable microenvironment first, BMSCs execute regeneration later.	CGRP effects not periodontium-specific; cell protection efficiency of porous microspheres not quantified.	Preclinical—small animal (periodontitis model)
Multifunctional microsphere	GM@Fe-Cur/PDA/MH injectable microspheres (Xiu Z. et al. 2025 [31])	Microfluidic GelMA microspheres; MH surface-adsorbed (PDA coating, electrostatic + π–π); Fe-Cur NPs matrix-encapsulated; spatial location → differential diffusion resistance → temporal release	Minocycline HCl (MH, antibacterial), Fe-Cur NPs (antioxidant + pro-osteogenic), PDA coating (NIR photothermal antibacterial)	MH: 57.80% cumulative at 96 h (60.08% in LPS). Fe-Cur NPs: only 6.96% at 96 h (7.28% in LPS). Early release rate difference: ~8-fold.	Fe-Cur NPs sustained ROS scavenging, macrophage polarization modulation, Nrf2 pathway activation, NLRP3 inflammasome inhibition.	Rat ligature-induced periodontitis: significantly reduced CEJ-ABC distance vs. untreated control (micro-CT).	Single carrier achieves ~8-fold release rate difference via surface loading vs. matrix encapsulation—no external trigger or multi-component assembly needed; PDA coating enables NIR synergistic antibacterial action.	Rat model only; surface-adsorbed MH may pose storage stability concerns.	Preclinical—small animal (periodontitis model)
Three-tier modular MN patch	Immunomodulatory MN patch (Zhang X. et al. 2022 [44])	Three-tier release logic: (1) gelatin baseplate rapid dissolution (~10 min) → immediate burst; (2) PLGA NPs (~150 nm) sustained release → days; (3) heparin-functionalized SiMPs (~15 μm, ~10 nm pores) → ~2 wk sustained release	Tetracycline HCl (antibacterial), IL-4 + TGF-β (immunomodulation)	Tier 1: ~10 min (gelatin dissolution). Tier 2: days (PLGA NPs). Tier 3: ~2 wk (SiMP degradation).	Complete bacterial growth inhibition; sustained IL-4/TGF-β ↓iNOS, IL-1β, ↑MRC1, ARG-1; Treg induction superior to soluble cytokines.	Rat periodontitis model: suppressed pro-inflammatory factors, promoted pro-regenerative signals and tissue healing.	Three kinetically distinct release tiers integrated into a single patch → modular multi-rate delivery; heparin modification dual function (enhanced loading + protein protection).	Multi-step fabrication (NPs + SiMPs + MN molding); PLGA NP release kinetics affected by in vivo pH/enzymes but not characterized.	Preclinical—small animal (periodontitis model)
MMP/pH dual-responsive core–shell MN	Core–shell MMP/pH dual-responsive MN (Liu J. et al. 2026 [38])	GelMA/SilMA shell with MMP-sensitive peptide sequences; SilMA core encapsulates MSC-Exo; MMP-9 cleavage → rapid GA-MOF release; exosomes subsequently released from core	GA-MOF (anti-inflammatory + antioxidant), MSC-Exo (immunomodulation + pro-osteogenic)	Shell GA-MOF: rapid MMP/pH-triggered release within first several days. Core MSC-Exo: sustained release over several weeks.	MSC-Exo induces M2 polarization (CD206^+^); BMSC osteogenic activity ↑2.3-fold (ALP) vs. untreated control.	Rat periodontitis model (d 28): CEJ-ABC distance↓, BV/TV↑, Tb.Th↑, RUNX2↑, OPN↑.	Enzyme + pH dual-trigger → release kinetics positively correlate with periodontitis severity; core–shell + dual-responsive combination.	Rat model only; inter-patient MMP variability → release kinetics predictability concerns.	Preclinical—small animal (periodontitis model)
Dual-nanozyme core–shell MN	Pd/Ce dual-nanozyme core–shell MN (Shan Y. et al. 2025 [45])	HA core (rapid dissolution) + MeHA shell (slow degradation); differential core–shell degradation rates → temporal release	Pd nanocubes (POD-like → ·OH bactericidal), DMSN-Ce nanozymes (DNase-like → NETs degradation)	Core Pd: 89.50% within 5 h. Shell DMSN-Ce: 87.35% over 7 d. Release timescale difference: ~33-fold.	Pd catalyzes H_2_O_2_ → ·OH (bactericidal); DMSN-Ce hydrolyzes NETs DNA backbone → suppresses NETs-driven tissue destruction.	Rat ligature-induced periodontitis: bacterial burden↓, NETs levels↓; micro-CT + histology: alleviated alveolar bone resorption.	Dual-nanozyme synergy (POD + DNase) → antibacterial + immunomodulation dual pathway; ~33-fold release timescale difference → clear temporal separation.	Rat model only; systemic toxicity/immunogenicity not evaluated; MeHA photocrosslinking batch variability.	Preclinical—small animal (periodontitis model)
B. Stimuli-Responsive Systems (Section 2.2)									
pH/ROS dual-responsive hydrogel	Ac2-26/OGP-loaded injectable hydrogel (Li R. et al. 2025 [62])	Dynamic boronate ester crosslinking; Ac2-26 linked via pH/ROS-sensitive boronate ester bonds → preferential cleavage; OGP encapsulated in insensitive GelMA framework → sustained release	Ac2-26 (Annexin A1 active fragment, anti-inflammatory/anti-pyroptosis), OGP (osteogenic growth peptide)	Ac2-26: >70% within 24 h in acidic + H_2_O_2_ environment. OGP: sustained steady release over several weeks.	Ac2-26 suppresses macrophage pyroptosis via ANXA1/NLRP3/caspase-1/GSDMD pathway + eliminates ROS.	Diabetic rat tooth extraction socket defect: sequential treatment → ~2.5-fold BV/TV increase.	Exploits dual pathological features of diabetic periodontitis (low pH + high ROS) for dual-lock release triggering; single injection; clear temporal separation (24 h vs. weeks).	Diabetic rat extraction socket only (not in situ periodontitis defect); OGP GelMA release kinetics affected by crosslinking density—not systematically characterized.	Preclinical—small animal (with metabolic disease model)
pH-evolution- driven dual-immuno- modulatory scaffold	DIBS (Zhao H. et al. 2026 [63])	Three-tier temporal logic: (1) OXG outer layer → pH-independent structural anti-inflammation → M1→M2; (2) low pH → Schiff base + metal coordination bond cleavage → Sr^2+^+PA accelerated release → MSC immune programming; (3) pH return to neutral → bond cleavage slows → HA framework mediates mineralization	Sr^2+^ (enhances MSC immunomodulation via CCL2/CCR2), PA (protocatechualdehyde, pro-osteogenic)	(1) M2 polarization: CD86↓/CD206↑ by d 3, M2 dominance by d 7. (2) Sr^2+^+PA acidic release: 1.8× and 1.25× vs. neutral over 20 d, peak aligned with MSC recruitment window (≤14 d post-injury). (3) Neutral pH: only ~15% mass loss over 20 d vs. 25–30% in acidic.	scRNA-seq: DIBS MSC subpopulation highly expresses CCL2, CCL7, CXCL1 → positioned at pseudotime trajectory origin; establishes macrophage–MSC positive-feedback loop.	Rat calvarial defect (12 wk): BV/TV = 23.97% new bone; CD31^+^Emcn^+^ Type H vessels↑.	Transforms complete pH dynamic evolution (acidic → neutral) into full closed-loop release program (activation → termination); extends sequential regulation from factor release to cell-behavior level; immune–vascular–osteogenic triple cascade.	Rat calvarial defect only (non-periodontitis); complex fabrication (3D-printed HA framework + hydrogel infusion + NP synthesis); long-term Sr^2+^ systemic distribution not tracked.	Preclinical—small animal
RgpA/ROS dual-responsive hydrogel	NHRT smart injectable hydrogel (Li X. et al. 2025 [77])	R8 peptide dual-responsive element: RgpA cleavage (threshold 1 nM) + ROS reaction (H_2_O_2_ threshold 10^−5^ M) → release TA + arginine + NO	Tannic acid (TA, antibacterial), Arginine (antibacterial), NO (anti-inflammatory, ROS-triggered only)	RgpA + H_2_O_2_: ~60% bacterial inhibition at 24 h (RgpA alone: ~23% at 12 h, ~41% at 24 h). NO released exclusively under ROS stimulation. Therapeutic logic follows natural pathological chronology: antibacterial first → anti-inflammatory later.	TA + arginine: rapid antibacterial; NO: targeted anti-inflammatory + immunomodulation.	Mouse periodontitis model (7 and 14 d): NHRT group significantly preserved alveolar bone vs. untreated control.	Virulence factor (RgpA) + inflammatory signal (ROS) dual-locking → therapeutic precision; R8 peptide serves as both RgpA substrate and ROS → NO converter.	Mouse model only; RgpA expression highly variable across patients/sites; local NO concentration–effect relationship not quantified.	Preclinical—small animal
pH/ROS dynamic boronate ester hydrogel	HTF@HA/CGF/BMP-2 injectable hydrogel (Qiu P. et al. 2026 [78])	Dynamic boronate ester crosslinking network; acidic + high ROS → boronate ester bonds preferentially hydrolyzed → TA rapid release; CGF intrinsic fibrin network → natural sustained release; HA adsorbs BMP-2 via Ca–phosphate interactions → ion-exchange sustained release	Tannic acid (TA, antioxidant + anti-inflammatory + NF-κB inhibitor), CGF (concentrated growth factors, multi-factor synergy), BMP-2 (osteogenic, low dose)	TA: rapid release in early acidic/high-ROS phase. CGF: sustained release (fibrin network). BMP-2: 79.36 ± 0.86% cumulative by d 18.	TA scavenges ROS, inhibits NF-κB → M2 polarization.	Rat periodontal defect (4 and 8 wk): low-dose BMP-2 (50 μg/L) group achieved bone regeneration comparable to clinical-standard high-dose group (500 μg/L, *p* > 0.05) → 10-fold BMP-2 dose reduction.	Converts pro-inflammatory microenvironment (pH↓ + ROS↑) into anti-inflammatory → osteogenic staged release program; substantial BMP-2 dose reduction → lower safety risk and cost.	Rat model only; autologous CGF → non-standardizable composition (inter-patient variability); boronate ester bond selectivity under multiple ROS species not verified.	Preclinical—small animal
NIR-triggered concentration- dependent	Hemin@ER-IR808 (Qiu X. et al. 2025 [80])	NIR irradiation (0.5 W/cm^2^, 30 s) → IR808 generates H_2_O_2_ → (1) direct bacterial killing; (2) opens erythrocyte membrane → high-concentration hemin release; hemin concentration decay → functional switch	IR808 (photosensitizer), Hemin (concentration-dependent functional switch)	No premature leakage for 5 d without NIR. NIR triggers immediate high-concentration hemin release; subsequent membrane/hydrogel slow degradation → hemin concentration decline.	High [hemin] → M1 polarization (glycolysis) + ferroptosis-like stress → bacterial killing. Low [hemin] → Nrf2/HO-1 + GPX4/SLC7A11 → M2 polarization + antioxidation.	Rat periodontitis model (2 and 4 wk): 2-wk CFU↓; 4-wk micro-CT alveolar bone resorption↓; maintained M2 immunomodulation + tissue repair after treatment cessation.	Single agent achieves functional transition via concentration-dependent biological switch → minimal active components; NIR provides externally controllable temporal trigger.	Erythrocyte membrane source → batch variability and immunogenicity; NIR penetration depth limited (~1 cm); clinical operability of intraoral NIR irradiation unproven.	Preclinical—small animal
NIR-triggered bilayer spatial separation	IP/C@SIS bilayer hydrogel (Ma S. et al. 2026 [81])	Outer SIS (10 mg/mL) loaded with IP (ICG@PCF-1); inner SIS (15 mg/mL) loaded with CeO_2_ NPs; outer layer degrades first → CeO_2_ exposed; NIR (808 nm, 1 W/cm^2^, 3 min) triggers photodynamic + photothermal effects	ICG (photosensitizer, PDT+PTT dual function), PCF-1 (HOF framework, enhances ICG photostability + ROS yield), CeO_2_ NPs (SOD+CAT-like, antioxidant)	IP 240 h cumulative: NIR(+) >70% vs. NIR(−) ~41.8%. CeO_2_: <8% cumulative within 13 d, sustained release after outer layer degrades. Outer layer nearly fully degraded by d 10.	NIR → IP generates ^1^O_2_ (bacterial killing) + local temperature rise to ~43 °C (synergistic antibacterial + accelerated outer degradation); CeO_2_ scavenges ROS + M2 polarization.	Rat ligature-induced periodontitis (42 d): IP/C@SIS+ group showed shortest CEJ-ABC distance and highest BV/CV ratio; near-complete eradication of Gram+ and Gram− bacteria; H&E+Masson: bone regeneration + PDL formation + mature collagen; heart/liver/spleen/lung/kidney H&E normal → excellent biosafety.	Two functional agents spatially isolated via bilayer structure → independently tunable release timing; NIR synergistically provides PDT+PTT dual-mode bacterial killing; SIS matrix intrinsically contains type I/III collagen + VEGF + FGF-2 → self-regenerative.	Rat model only; long-term in vivo stability of bilayer interlayer interface unverified; NIR requires multiple irradiation sessions (0/24/48/96/144 h) → clinical convenience limited.	Preclinical—small animal (periodontitis model)
C. Cell/EV-Based Systems (Section 2.3)									
M2 macrophage exosome engineering	Mel@M2-exos (Cui Y. et al. 2023 [85])	(1) M2-exos inflammatory tropism → active recognition + preferential uptake by M1 macrophages; (2) M2-exos membrane fusion + nucleic acid delivery → host M1→M2 reprogramming; (3) intracellular exosome degradation → melatonin sustained release → ER stress modulation → osteogenesis/cementogenesis	Melatonin (ER stress modulation + anti-apoptotic), M2-exos intrinsic cargo (proteins + nucleic acids, immune reprogramming)	In vivo tracing: DiD-M2-exos reached periodontal inflammatory site by 8 h, peak enrichment at 24 h, gradually fading. Melatonin: sustained release upon intracellular exosome degradation.	LPS-activated inflammatory macrophages internalize M2-exos at significantly higher levels than non-activated cells; melatonin ↓GRP78, CHOP; modulates PERK/ATF6/IRE1 → ↓caspase-3, ↑Bcl-2 → breaks ER stress–apoptosis vicious cycle → restores ALP, COL1, Runx2.	Rat periodontitis model (GelMA hydrogel carrier): significantly inhibited alveolar bone resorption; closed-loop sequential therapy from immunomodulation to tissue regeneration.	Inflammatory tropism → passive targeting to lesion core; dual-mode immune reprogramming (protein transfer + transcriptional reprogramming); non-living nanoparticle (distinct from live-cell therapy).	Only one principal system discussed (Section 2.3.1); low exosome biogenesis efficiency; batch variability + cold-chain dependence + no universal potency assay → greatest manufacturing hurdles (see Section 2.3.2); no large-animal data.	Preclinical—small animal. Longest manufacturing + regulatory pathway (see Section 2.3.2)
D. Asymmetric Structural Design (Section 2.4)									
Asymmetric membrane + ROS-responsive coating	PBMC (Chen E. et al. 2024 [89])	LbL self-assembly of polyphenol-based intelligent coating (EPi-gallocatechin gallate + Minocycline) on collagen barrier membrane; retains traditional GBR dual-sided structure (smooth side blocks epithelium + rough side promotes cell attachment) + introduces ROS-responsive boronate ester bond coating	Minocycline (antibacterial), Natural polyphenols (sustained ROS scavenging)	ROS-triggered controlled rapid minocycline release (early phase); polyphenols continuously scavenge ROS (throughout). Specific release time window not quantified in original paper.	PBMC scavenges intracellular ROS, protects PDLSCs from H_2_O_2_-induced oxidative damage; enhances PDLSC migration and osteogenic differentiation (ALP↑, ARS↑).	Rat periodontal bone defect + postoperative *P. gingivalis* infection model (40 SD rats, 6–8 wk): EBMC/OBMC groups showed significantly reduced CEJ-ABC distance vs. COL and control at 4 and 6 wk; BV/TV and Tb.N significantly higher, Tb.Sp lower; 6-wk H&E+Masson: significant new alveolar bone (NAB) + neovascularization + collagen matrix; IHC: RUNX-2 and OCN significantly elevated (*p* < 0.0001).	Upgrades traditional GBR static spatial partitioning to molecular-level temporal functional synergy; based on mature collagen membrane platform → better translational foundation; validated in infection model (more clinically realistic).	Rat model only; long-term in vivo stability of ROS-responsive coating unverified; quantitative release curve not provided; precise polyphenol/minocycline loading amounts not quantified.	Preclinical—small animal (periodontal defect + infection model)
Hierarchical nanotopography + biochemical signals	TS@BMP-2 (He Z. et al. 2024 [90])	Coaxial electrospinning core–shell fiber + PCL shell surface-directed epitaxial crystallization → cyclic nano-lamellar (coral-reef-plate-like) topography; early command: topographic physical signal → NF-κB inhibition → M2 polarization; late command: BMP-2 sustained release from PVA core	Nano-topographical cues (physical signal, immunomodulation), BMP-2 (osteogenic)	Early: topographic signal immediate at implantation (no trigger/degradation dependence). Late: BMP-2 sustained release from core (core–shell provides time lag). Specific release time window not quantified in original paper.	Nano-topography directly modulates macrophages → M2 polarization (NF-κB pathway inhibition); BMP-2 → BMSC osteogenic differentiation + mineralization.	Mouse periodontitis model (1 and 4 wk): TS@BMP-2 group exhibited best BV/TV and Tb.N, lowest Tb.Sp; shortest CEJ-ABC distance (least alveolar bone resorption); H&E: increased osseous tissue; Masson: denser PDL + increased collagen; IHC: lowest IL-1β, fewest TRAP^+^ osteoclasts; highest osteogenic activity at 4 wk.	Spatiotemporal coordination of physical + biochemical signals—material structure-inherent logic (not environmentally passive); physical signal durable (degradation-independent, enzyme/ROS-independent); topography simultaneously inhibits inflammation + bone resorption → dual protection.	Mouse model only; nano-topography fabrication (surface-directed epitaxial crystallization) has narrow parameter window; synergistic vs. additive effects of BMP-2 + topography not dissected.	Preclinical—small animal (periodontitis model)

## Data Availability

No new data were created or analyzed in this study. Data sharing is not applicable to this article.
